# A Web-Based Health Promotion Program for Older Workers: Randomized Controlled Trial

**DOI:** 10.2196/jmir.3399

**Published:** 2015-03-25

**Authors:** Royer F Cook, Rebekah K Hersch, Dana Schlossberg, Samantha L Leaf

**Affiliations:** ^1^ISA AssociatesAlexandria, VAUnited States

**Keywords:** Internet, health promotion, middle aged, nutritional requirements, exercise

## Abstract

**Background:**

Recent evidence supports the efficacy of programs that promote improvements in the health practices of workers 50 years and older who are at higher risk for chronic diseases than younger workers are. Internet-based programs that promote healthy practices have also shown promise and, therefore, should be especially appropriate for workers aged 50 years and older.

**Objective:**

The purpose of the research was to evaluate the effectiveness of HealthyPast50, a fully automated Web-based health promotion program based on social cognitive theory and aimed specifically at workers 50 years and older.

**Methods:**

The randomized controlled trial was conducted across multiple US offices of a large global information technology company. The sample included 278 employees aged 50 to 68 who were recruited online and randomly assigned to the Web-based HealthyPast50 program or to a wait-list control condition. Self-report measures of diet, physical activity, stress, and tobacco use were collected online before and 3 months after the program group was given access to the program. Use data included number of log-ins and number of pages accessed. The primary analysis was multiple linear regression, following intent-to-treat principles with multiple imputation using the Markov chain Monte Carlo (MCMC) approach for nonmonotone missing data. Potential moderators from demographic characteristics and program dosage effects were assessed using multiple linear regression models. Additional analyses were conducted on complete (nonimputed) cases, excluding program participants who used the program for less than 30 minutes.

**Results:**

Retention rates were good for both groups: 80.4% (111/138) for the program group and 94.3% (132/140) for the control group. Program group participants spent a mean of 102.26 minutes in the program (SD 148.32), logged in a mean of 4.33 times (SD 4.28), and viewed a mean of 11.04 pages (SD 20.08). In the analysis of the imputed dataset, the program group performed significantly better than the control group on diet behavioral change self-efficacy (estimated adjusted difference [Δ]=0.16, *P*=.048), planning healthy eating (Δ=0.17, *P*=.03), and mild exercise (Δ=1.03, *P*=.01). Moderator and dosage analyses of the dataset found no significant program effects. Analyses of the nonimputed dataset comparing program users with controls found additional significant program effects on eating practices (Δ=0.09, *P*=.03), exercise self-efficacy (Δ=0.12, *P*=.03), exercise planning (Δ=0.18, *P*=.03), and aging beliefs (Δ=0.17, *P*=.01). Moderator analysis of this dataset also found significant moderator effects of gender on multiple measures of exercise.

**Conclusions:**

A Web-based health promotion program showed promise for making a significant contribution to the short-term dietary and exercise practices of older working adults. Gender effects suggest that the program effects on exercise are due mainly to improvements among women.

## Introduction

Decades of studies have confirmed the link between chronic illness and common modifiable risk factors, such as smoking, physical inactivity, poor diet, and high stress [1,2]. Moreover, there is ample evidence that the large and expanding group of older workers—a sizable group numbering more than 50 million people—are at higher risk for costly chronic diseases than their younger coworkers [3]. It has also been shown that if workers in midlife can reduce their modifiable health risks, they can forestall disability and reduce their utilization of health care services. By doing so, they can increase the likelihood of a healthful, enjoyable life in their later years while also contributing to a possible reduction in health care costs [3-5].

Consequently, providing older workers (50 and older) with effective tools that can help them improve their health practices could be beneficial to these workers, who are more likely to be affected by major diseases such as cancer, cardiovascular disease, diabetes, and musculoskeletal disease. Moreover, decreasing the health risks of older workers has special relevance for employers and health care companies because annual losses from chronic diseases exceed US $1 trillion (including productivity losses), and older workers account for a disproportionate share of the organization’s health care costs [6]. In addition, from a public policy perspective, there is a particularly strong incentive for reducing the health risks of workers in this group because they will be moving onto the Medicare rolls at age 65, swelling the enrollee population to more than 70 million by 2030. There is ample evidence that well-constructed health promotion programs for the workplace can be excellent mechanisms for increasing worker health, decreasing health care costs, and improving productivity [7-9]. Moreover, there is accumulating evidence that health promotion programs developed specifically for older adults, with their distinctive set of health risks and age-appropriate health practices (eg, focusing on regular moderate activity rather than highly aerobic exercise), can significantly improve their health practices and lower their health risks [10-13].

Computer-based approaches to workplace health promotion and disease prevention strategies have become more numerous, including Web-based modes of delivery [14-22]. With the advent of broadband, high-speed connections (especially prevalent in workplaces), Web-based programs offer the advantages of tailored, media-rich psychoeducational experiences accessible at any time or place where an Internet connection is available. Research on Web-based programs indicates that such approaches can be an effective means of contributing to positive changes in diet, physical activity, stress management, and substance misuse [14-19]. However, with the exception of the study by Hughes and associates [11], none of the more promising studies focused on older working adults, but drew their samples through Internet recruiting [16,18] and church groups [15].

Because there are physical and psychological characteristics of older workers that distinguish them from younger workers, any workplace intervention directed at this older group must be tailored to their particular needs and characteristics. Most of the health promotion topics that comprise the typical workplace health promotion program (eg, promoting physical activity, healthy eating, stress management) should also be part of an older workers’ program. However, both the content of the programs and the recruitment and motivational strategies would most appropriately be altered for this age group.

The purpose of the research was to evaluate the impact of a multimedia Web-based health promotion program on central health attitudes and practices of older workers. This program, called HealthyPast50, was designed as a stand-alone intervention to address a wide variety of health behavior topics, including physical activity, healthy eating, stress management, and tobacco cessation—the health behaviors that contribute to the prevention of major diseases.

## Methods

### Design

The impact of a multimedia Web-based program on the health practices of workers 50 years of age or older was assessed in a randomized controlled trial (RCT) in which participants were randomly assigned to the Web-based program condition or to a wait-list control condition. Participation was voluntary and all protocols and procedures were approved by the ISA Associates, Inc Institutional Review Board, Alexandria, VA. All participants, employees of a large global information technology company, were surveyed on multiple outcome measures before and 3 months after the program group received access to the Web-based program. The specific objectives of the study were to assess the extent to which the Web-based program produced significant positive changes, from baseline to posttest, on measures of stress, diet, physical activity, aging beliefs, and tobacco use. All outcome measures were self-reports, collected through an online survey. The full study protocol is shown in Multimedia Appendix 1.

### Procedures

A recruitment flyer briefly describing the purpose of the study was emailed by company officials to all employees 50 years of age and older (approximately 2500 employees) located in multiple US offices of a global information technology company. The flyer stated that the study was being conducted by a research organization through a grant from the National Institutes of Health. The flyer also explained that participants would receive US $25 for completing the first questionnaire and US $25 for completing the second questionnaire, and that their name would be entered into a drawing in which 1 participant would receive US $500 during each questionnaire round. Interested employees who fit the inclusion criteria (age 50 and older) were instructed to contact the project staff directly by email or telephone. When interested employees contacted the study team, they were provided additional information about the study and their eligibility to participate (ie, age and employment at the company) was confirmed. Employees interested in participating after learning more about the project provided the study team with an email address to be used to send a personalized email and link to the online baseline survey, which also included the consent document. The full consent document is shown in Multimedia Appendix 2.

After reading the consent document, participants selected 1 of 2 responses indicating whether they consented or declined to participate. Participants were not able to continue with the survey until they acknowledged and indicated that they consented to participate. No participant declined to participate (although, as noted subsequently, some initially interested employees did not complete the baseline survey and 1 employee withdrew after consenting to participate and completing the survey). An electronic copy of the consent document was then emailed to participants after they completed the baseline survey.

**Figure 1:**
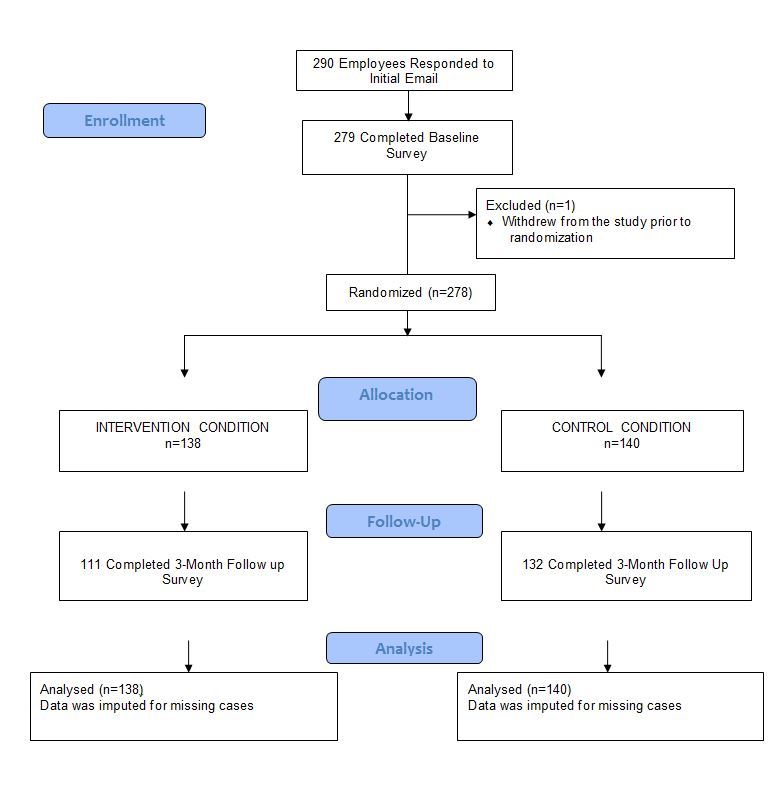
Participant flow diagram.

A total of 290 employees contacted the study team in response to the initial email and were sent a link to the online survey, 279 of whom completed the baseline survey. One participant subsequently contacted the study team to withdraw from the study leaving a total sample size of 278 participants (Figure 1).

Randomization was conducted by the second author using a block-randomized design with blocks of 4 and 6. The 0 and 1 within each block were random and the order of the group of 4 and the group of 6 was random. Randomization occurred after each participant completed the baseline survey. The online survey program was checked every day to determine who completed the survey each day and individuals were assigned to the next condition on the randomization table as they completed the survey. Once randomization was complete, participants were notified of the condition to which they were assigned (no blinding procedures were employed) and were informed of next steps; the program group was given the program link and log-in information and the control group was told that their access to the program would be delayed until the end of the test period. Participants could complete the online questionnaires and (for those in the program condition) access the online program on work time or at home.

Participants in the program group could access the Web-based program at any time during the 3-month test period, both at work or outside work (eg, at home). The program operated alone and automatically; no human contact was involved. A “project update” email was sent to participants at 1 month and 2 months after randomization. For the program group, the emails included a reminder to use the program and information about the latest update to the program (a brief message on the home page). With the exception of the project update, the program was unchanged throughout the test period. For the control group, the email included information about when the second survey would be available. In addition, the project staff was always available to answer questions by telephone and email if participants had any difficulty accessing the program. The data collection started on October 10, 2012 (first participant enrolled) and ended on February 23, 2013 (last participant completed posttest). Individual access to program by participants was limited to the 3-month test period. No discernable secular events of note occurred during the test period. At the end of the 3-month test period and prior to administering the follow-up survey, access to the program was blocked. Three months after randomization, participants were sent an email with the link to the follow-up survey. After the posttest survey was complete, all participants received access to the program.

### Sample

Study participants were 278 employees of a large global information technology company whose US offices are located primarily in Massachusetts and California. The study was powered for a sample size of 250 (after attrition) based on previous studies by the authors. The computer literacy of the workforce was estimated to be relatively high. Demographic characteristics of the sample are displayed in Table 1. Participants ranged in age from 50 to 68 years, 67.3% (187/278) of whom were male and 89.6% (249/278) identified as Caucasian. Participants were relatively affluent and well educated: 64.7% (180/278) of participants had a Bachelor’s degree or higher and 70.1% (195/278) reported a household income of US $100,000 or more. A comparison of the program group with the control group on demographics and outcome variables at baseline revealed no significant differences between the groups indicating that randomization was successful.

**Table 1:** Participant characteristics at baseline.

Demographics		Control, n (%) n=140	Program, n (%) n=138	Total, n (%) N=278
**Gender**				
	Male	89 (63.6)	98 (71.0)	187 (67.3)
	Female	50 (35.7)	40 (29.0)	90 (32.4)
	Prefer not to answer	1 (0.7)	0	1 (0.4)
**Race**				
	Black or African American	6 (4.3)	3 (2.2)	9 (3.2)
	Caucasian	123 (87.9)	126 (91.3)	249 (89.6)
	American Indian/Alaska Native	1 (0.7)	0	1 (0.4)
	Asian	5 (3.6)	2 (1.4)	7 (2.5)
	Native Hawaiian or Other Pacific Islander	1 (0.7)	1 (0.7)	2 (0.7)
	Multiracial	2 (1.4)	4 (2.9)	6 (2.2)
	Prefer not to answer	2 (1.4)	1 (0.7)	3 (1.1)
**Latino**				
	Yes	4 (2.9)	4 (2.9)	8 (2.9)
	No	134 (95.7)	133 (96.4)	267 (96.0)
	Prefer not to answer	2 (1.4)	1 (0.7)	3 (1.1 )
**Age**				
	50-54	74 (52.9)	64 (46.4)	138 (49.6)
	55-59	36 (25.7)	47 (34.1)	83 (29.9)
	60-64	22 (15.7)	22 (15.9)	44 (15.8)
	65-69	8 (5.7)	5 (3.6)	13 (4.7)
**Marital status**				
	Single	6 (4.3)	9 (6.5)	15 (5.4)
	Married	114 (81.4)	101 (73.2)	215 (77.3)
	Divorced	14 (10.0)	16 (11.6)	30 (10.8)
	Separated	0	5 (3.6)	5 (1.8)
	Widowed	2 (1.4)	3 (2.2)	5 (1.8)
	Living with a partner	4 (2.9)	4 (2.9)	8 (2.9)
**Income (US $)**				
	<$60,000	4 (2.9)	6 (4.3)	10 (3.6)
	$60,000-$79,999	7 (5.0)	12 (8.7)	19 (6.8)
	$80,000-$99,999	15 (10.7%)	11 (8.0)	26 (9.4)
	$100,000 or more	102 (72.9)	93 (67.4)	195 (70.1)
	Prefer not to answer	12 (8.6)	16 (11.6)	28 (10.1)
**Education**				
	High school diploma or equivalent	8 (5.7)	6 (4.3)	14 (5.0)
	Vocational/technical training after high school	5 (3.6)	12 (8.7)	17 (6.1)
	Some college, but no degree	22 (15.7)	19 (13.8)	41 (14.7)
	Associate’s degree (AA, AS)	12 (8.6)	14 (10.1)	26 (9.3)
	Bachelor’s degree (BA, BS, AB)	45 (32.1)	43 (31.2)	88 (31.7)
	Graduate or professional school, but no degree	10 (7.1)	13 (9.4)	23 (8.3)
	Master’s degree (MA, MS)	34 (24.3)	28 (20.3)	62 (22.3)
	Doctorate degree (PhD, EDD)	4 (2.9)	2 (1.4)	6 (2.2)
	Professional degree beyond Bachelor’s degree (MD, DDS, JD)	0	1 (0.7)	1 (0.4)

### Intervention

**Figure 2:**
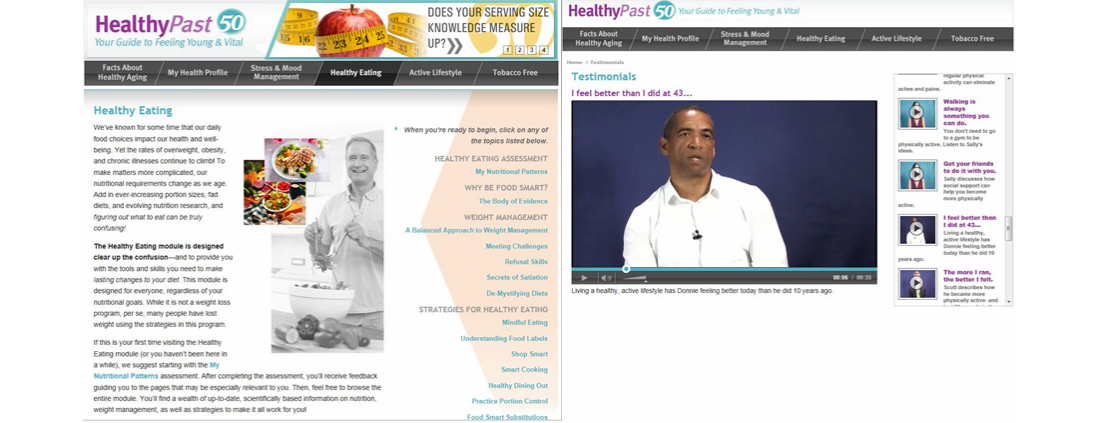
Screenshots from HealthyPast50.

**Figure 3:**
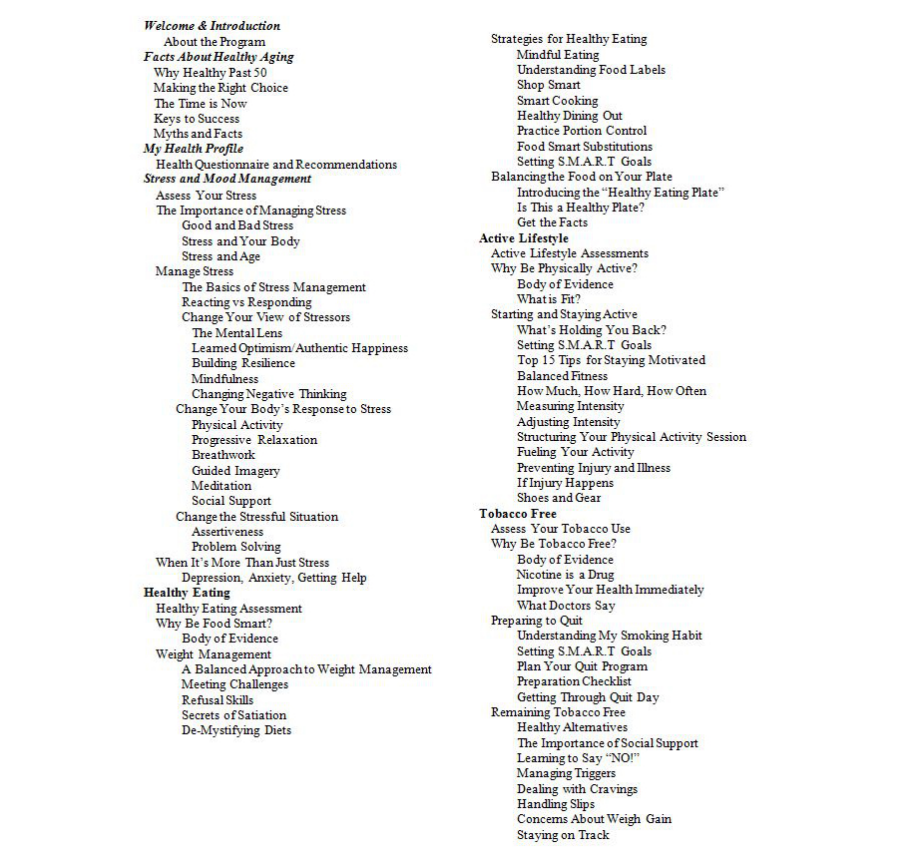
HealthyPast50 content outline.

HealthyPast50 is a Web-based multimedia program containing information and guidance on the major health promotion topics of healthy aging, diet, physical activity, stress management, and tobacco use. In addition, a central module of the program contained assessments across the major health topics, providing recommendations on particular segments of HealthyPast50 that users should visit based on the results of the assessments. Sample screenshots are shown in Figure 2 and in Multimedia Appendix 3. An outline of program content is shown in Figure 3. On the home page, users were encouraged to complete the assessments before accessing the other modules. These recommendations were the only form of tailoring used in the program. With the exception of the module on healthy aging, all the major modules incorporated material from previously developed and tested programs from our group (for which evidence of efficacy was shown), modifying the content and approaches for the 50 and older audience [19,22-23].

Program content was shaped specifically for older adults in many ways. Throughout the program, from the home page through all the modules, there are numerous depictions (eg, photos, graphics) of older adults along with dozens of video segments (testimonials) featuring people older than 50 years of age describing their health practices, struggles, and successes. An entire module is devoted to “Facts About Healthy Aging,” emphasizing the importance of making positive changes in health practices now, and containing a segment on “Aging Myths and Facts.” The assessment module (My Health Profile) is geared to older adults, tailored by age (eg, 50-59, 60-69) and including questions about current diseases and preventive health screenings. A central segment of the Stress and Mood Management module describes with text, video, graphics, and narration, the relationship between stress and aging, featuring detailed information on how stress accelerates the aging process at the cellular level by shortening the ends of chromosomes (telomeres). Dietary information in the Healthy Eating module emphasizes the role of a nutritious diet and a healthy weight in preventing chronic disease and increasing longevity. Much of the content of the Active Lifestyle module is oriented toward older users, including the use of the Physical Activity Readiness Questionnaire (PAR-Q), assessing one’s readiness for physical activity, the relationship of physical activity to mobility and energy in later years, and the need to moderate the intensity of exercise as one ages.

The program functions were monitored throughout the test period to ensure quality of operation. As with our group’s previous Web-based interventions, HealthyPast50 was shaped by a social-cognitive conceptual model based primarily on the work of Bandura [24-25], emphasizing the boosting of self-efficacy, self-regulation, and planning. A central premise of this model is that to achieve lasting improvements in health behavior, an intervention must do more than provide information about a given health topic; it must also provide the skills and motivation that are essential to making lasting improvements in one’s health practices.

The Web-based HealthyPast50 program was developed by our group over a 2-year period through multiple cycles of development and testing, beginning with focus groups of older workers (age 50 and older) providing feedback on specific features of the planned program, followed by ratings of prototype content, and culminating in the workplace-based RCT. The program was constructed using the ColdFusion-based Mura content management system, with the many interactive elements and assessments developed in Flash. The program also contains ample graphics, audio, and video. Many of these main elements are congruent with health behavior change theory and principles (eg, providing opportunities for observational learning, building self-efficacy, and self-tailoring of content and sequence).

For access to the full HealthyPast50 program for review or replication purposes, please contact the first author.

### Measures

#### Overview

The 30-45 minute online self-report survey contained the measures described subsequently. In addition, program utilization data, including number of log-ins, minutes in the program, and pages accessed, were recorded for each member of the program group.

#### Demographics

Seven items assessing respondents’ gender, age, race, ethnicity, marital status, education, and income.

#### Symptoms of Distress

A 15-item scale developed by Orioli et al [26] and used in multiple studies by our team [19,27]. Each item describes a physical or emotional symptom of distress (eg, muscle tension, nervousness) with a 4-point response scale indicating the frequency with which the symptom was felt in the past 30 days, ranging from 1 (nearly every day) to 4 (never); a higher score=less stress (α=.85).

#### Coping With Stress

Twelve items assessing the type of strategies one uses to cope with difficult situations and events [26]. Questions are answered on a 4-point scale ranging from 1 (never) to 4 (almost always); higher score=better coping. Typical questions included “I often put things aside for a while to get perspective on them” and “I decide certain problems are not worth worrying about” (α=.76).

#### Diet Outcome Expectancies

A 9-item scale developed and validated by Trenkner and associates [28] assessing perceived benefits to eating a healthy diet. The response scale is a 5-point Likert scale ranging from 1 (strongly disagree) to 5 (strongly agree); a higher score=higher perceived benefits. Typical items included “Eating a poor diet increases my chances of getting diseases like heart disease, cancer, and diabetes” and “Eating more fruits and vegetables will make me healthier” (α=.80).

#### Barriers to a Healthy Diet

An 8-item scale also developed and validated by Trenkner and associates [28], assessing perceived barriers to eating a healthy diet. The response scale is a 5-point Likert scale ranging from 1 (strongly disagree) to 5 (strongly agree); a higher score=lower perceived barriers. Typical items included “It’s easy to buy healthy foods in a grocery store” and “A lot of things get in the way of my eating a more healthy diet” (α=.82).

#### Eating Practices

A 10-item subscale that is part of the Weight Control Assessment scale developed by O’Neil and Rhodes [29], the measure assesses the frequency with which respondents exercise control over their eating during the past 30 days. The response scale is a 4-point Likert scale ranging from 1 (almost always) to 4 (never); a higher score=better control over eating. It contained items such as “How often do you eat between meals” and “Do you have trouble controlling your eating when your favorite foods are around the house?” (α=.77).

#### Overeating Self-Efficacy

A 15-item scale assessing one’s confidence in resisting overeating in different situations, with responses on a 7-point scale ranging from 1 (no difficulty controlling overeating) to 7 (most difficulty controlling overeating). Typical items included “overeating when depressed” and “overeating around holiday time.” Developed by McCann et al [30], this scale is a shortened version of the 25-item Eating Self-Efficacy Scale [31]. A lower score indicates greater self-efficacy (α=.95).

#### Diet Change Self-Efficacy

A 5-item scale previously used by the study team [22] that asks respondents how confident they are that they can change their dietary practices. The response scale has 5 points, from 1 (not confident) to 5 (extremely confident); a higher score=higher self-efficacy. Typical items included “How confident are you that you have the skills to eat a healthy diet?” and “How confident are you that you have the skills to eat more fruits and vegetables?” (α=.88).

#### Planning Healthy Eating

A 2-item scale that asks respondents if they have a good plan for “maintaining a nutritious diet” and for “minimizing the amount of fats and sugars in my diet.” The response scale for both items is a 4-point scale, ranging from 1 (not at all true) to 4 (definitely true); a higher score=better planning (item *r*=.77).

#### Weight and Body Mass Index

Two items ask respondents to report their height and weight, yielding both weight and body mass index (BMI) measures.

#### Exercise Habits

The Godin Leisure-Time Exercise Questionnaire [32] is a brief 4-item query of usual leisure-time exercise habits, generating 5 activity scores. The first 3 items ask respondents to indicate the times per week they engage in strenuous, moderate, and mild exercise; the 3 items are combined to yield a score for overall exercise. The fourth item (the “sweat” score) asks how often the respondent engages in an activity long enough to work up a sweat; the 3-point response scale ranges from 1 (often) to 3 (never/rarely). A lower sweat score is better. Reliability and concurrent validity of the measure were demonstrated by Godin and Shephard [32].

#### Exercise Self-Efficacy

An 8-item scale assessing the respondent’s confidence in engaging in regular exercise. All items begin with “I am confident that...” and are answered on a 4-point response scale from 1 (not at all true) to 4 (always true); a higher score=higher exercise self-efficacy. Typical items included “I can accomplish the physical activity and exercise goals that I set” and “I can overcome barriers to and challenges to physical activity and exercise if I try hard enough.” This scale was developed by Kroll et al [33] (α=.91).

#### Self-Efficacy for Overcoming Barriers to Exercise

A 13-item scale that asks respondents how confident they are that they can “exercise 3 times a week for the next 3 months” under potentially difficult circumstances, including bad weather, lack of interest, schedule conflicts, etc. The 10-point response scale ranges from 0 (not at all confident) to 100 (highly confident) in 10-point intervals; a higher score=higher self-efficacy to overcome barriers to regular exercise. This scale was developed by McAuley [34] (α=.94).

#### Exercise Planning

A 2-item scale that asks respondents if they have a good plan for “incorporating regular physical activity into my life” and “overcoming barriers to getting regular physical activity.” The response scale for both items is a 4-point scale, ranging from 1 (not at all true) to 4 (definitely true); a higher score=a better plan (item *r*=.85).

#### Beliefs About Aging

A 5-item scale that asks respondents about the extent to which they hold healthful attitudes about aging. Typical items include “The slowing of metabolism with age makes it even more important to eat a nutritious and well-balanced diet” and “Physical and mental decline is a natural part of the aging process, and you really can’t slow it down” (reverse scored). The response scale is a 5-point Likert scale ranging from 1 (strongly agree) to 5 (strongly disagree); a higher score=more healthful attitudes (α=.54).

#### Tobacco Use

Seven items assessed whether or not participants currently smoke cigarettes or use other tobacco products and, if so, how often they use tobacco products, how many times they use tobacco products per day, and whether and how often they have tried to quit using tobacco products.

### Analysis

Multiple linear regression models were used to examine program effects on outcome measures and potential moderators and to assess dosage effects. Analyses followed intent-to-treat principles, including all participants irrespective of protocol violations and events arising postrandomization [35]. Multiple imputation was conducted for missing values of all outcome variables, using the Markov chain Monte Carlo (MCMC) approach for nonmonotone missing data [36]. Data used to construct the imputation models were baseline demographic characteristics (eg, gender, age, race, marital status, education, and income), group assignment, and the corresponding baseline (pretest) measures. The program effects, moderators, and program dosage effects for each outcome variable were assessed using multiple linear regression models after adjusting for the baseline measure, based on 20 imputed datasets. SAS Version 9.3 (SAS Inc, Cary, NC, USA) was used to perform each analysis (PROC GLM for the regression models; PROC MI and MIANALYZE for the multiple imputation).

On most outcome variables, missing data occurred for less than 15% of the participants. Before imputing missing data, attrition analysis was conducted to examine potential baseline differences between those who completed the follow-up survey and those who did not. These data indicated that responders and nonresponders were virtually equivalent. Chi-square tests showed no differences between the groups on age (*P=*.41), gender (*P=*.99), race (*P=*.38), income (*P=*.80), or education (*P=*.33). Comparing the 2 groups on all outcome measures at baseline revealed significant differences only on symptoms of distress (*t*_269_=2.53, *P*=.01).

To assess program effects on program users, we also conducted the same multiple linear regression analysis on a nonimputed dataset that excluded participants in the program group who used the program for less than 30 minutes.

## Results

### Program Effects on Primary Outcomes

Table 2 presents the results of the estimated adjusted posttest difference between program and control groups on measures of diet, exercise, and stress.

The program group showed significantly greater improvement than the control group on diet behavioral change self-efficacy (estimated adjusted difference [Δ]=0.16, *P=*.048), and planning healthy eating (Δ=0.17, *P*=.03). The estimated adjusted difference [Δ] for eating practices was 0.07 and was not statistically significant (*P*=.08). In addition, there were no differences between the groups on diet outcome expectancies, healthy diet barriers, or overeating self-efficacy.

Compared to the control group, the program group showed significant improvement on mild exercise (Δ=1.03, *P*=.01). Two other measures of physical activity, including moderate exercise (Δ=0.47, *P*=.06) and overall exercise (Δ=4.98, *P*=.08) were not statistically significant. In addition, there were no differences between the groups on strenuous exercise, sweat, exercise self-efficacy, exercise planning, or self-efficacy for overcoming barriers to exercise. There were no differences between the program and control groups on symptoms of distress or coping with stress. However, the lack of a significant effect may be partly a function of a ceiling effect (ie, the mean scores of both groups at baseline indicated that participants in both groups entered the test period with relatively low stress and high coping skills).

There was also no significant difference between the groups on the measure of aging beliefs, although the trend was in the desired direction (Δ=0.09, *P*=.16).

There were insufficient numbers of smokers in the sample (only 16 smokers in the sample) to perform meaningful analysis on the measure of tobacco use.

**Table 2:** Adjusted program effects on dependent measures

Measure		Estimated adjusted posttest difference between program and control groups,^a^ Δ (95% CI)	*P*
**Eating and diet measures**			
	Diet outcome expectancies	0.02 (–0.08, 0.11)	.76
	Healthy diet barriers	0.05 (–0.07, 0.17)	.43
	Eating practices	0.07 (–0.01, 0.15)	.08
	Overeating self-efficacy	–0.14 (–0.36, 0.08)	.20
	Diet change self-efficacy	0.16 (0.00, 0.31)	.05
	Planning health eating	0.17 (0.01, 0.33)	.03
	BMI	0.07 (–0.28, 0.41)	.70
**Exercise measures**			
	Godin: Strenuous exercise	–0.11 (–0.52, 0.31)	.61
	Godin: Moderate exercise	0.47 (–0.01, 0.96)	.06
	Godin: Mild exercise	1.03 (0.26, 1.81)	.01
	Godin: Sweat	0.08 (–0.08, 0.23)	.33
	Godin: Overall exercise	4.98 (–0.66, 10.62)	.08
	Overcoming barriers to exercise self-efficacy	–0.68 (–5.55, 4.19)	.78
	Exercise self-efficacy	0.08 (–0.02, 0.18)	.11
	Exercise planning	0.11 (–0.04, 0.25)	.15
**Stress and coping measures**			
	Symptoms of distress	0.05 (–0.03, 0.13)	.22
	Coping with stress	0.01 (–0.05, 0.07)	.79
	Aging beliefs	0.09 (–0.03, 0.21)	.16

^a^ Adjusted for the corresponding baseline measure.

### Moderator and Dosage Effects

To determine whether program effects on outcomes differed based on participant demographics, potential moderators were tested on all outcome measures. Regression analysis models included main effects as well as interactions between condition and potential moderators. No significant interactions were detected between condition and gender, age, marital status, education, or income on any outcome measures.

Data on number of log-ins, minutes in the program, and number of pages accessed were recorded for all members of the program group. An examination of the distribution of number of minutes in the program revealed one major outlier of 2053 minutes (more than 34 hours), more than twice as long as the next longest number of minutes. In calculating average use of the program, this participant’s data were removed. The mean number of log-ins was 4.33 (SD 4.28, range 0-28), the mean number of minutes in the program was 102.26 minutes (SD 148.32), and the mean number of pages viewed was 11.04 (SD 20.08, range 0-120). An examination of the distribution of number of minutes in the program showed that 39 participants spent less than 30 minutes in the program and 99 participants—71.7% (99/138) of the program group—spent 30 minutes or more in the program.

To assess the extent to which the program effects were associated with the extent to which participants in the program group accessed the HealthyPast50 program, multiple regression analysis was conducted to examine the dosage effect of the number of pages viewed on all outcome variables. There were no significant associations between the number of pages viewed and any of the outcome measures after adjusting for the corresponding baseline measures.

### Program Effects Excluding Nonusers

A total of 39 participants in the program group who used the program less than 30 total minutes were defined as “nonusers” and were excluded from the analysis of the nonimputed dataset. The results of these analyses are shown in Table 3. In addition to the significant program effects found in the analysis of the imputed dataset, the analysis of the nonimputed dataset with nonusers excluded found significant program effects on eating practices (Δ=0.09, *P*=.03), exercise self-efficacy (Δ=0.12, *P=*.03), exercise planning (Δ=0.18, *P=*.03), and aging beliefs (Δ=0.17, *P=*.01).

**Table 3:** Adjusted program effects on dependent measures comparing legitimate program users and controls using complete cases.

Measure		Estimated adjusted posttest difference between program and control groups,^a^ Δ (95% CI)	*P*
**Eating and diet measures**			
	Diet outcome expectancies	–0.01 (–0.12, 0.10)	.85
	Healthy diet barriers	0.06 (–0.06, 0.19)	.34
	Eating practices	0.09 (0.01, 0.18)	.03
	Overeating self-efficacy	–0.24 (–0.47,–0.00)	.047
	Diet change self-efficacy	0.18 (0.02, 0.34)	.03
	Planning health eating	0.24 (0.07, 0.40)	.01
	BMI	0.05 (–0.38, 0.47)	.83
**Exercise measures**			
	Godin: Strenuous exercise	–0.09 (–0.55, 0.36)	.68
	Godin: Moderate exercise	0.35 (–0.23, 0.92)	.24
	Godin: Mild exercise	0.78 (–0.06, 1.62)	.07
	Godin: Sweat	0.03 (–0.14, 0.21)	.70
	Godin: Overall exercise	3.43 (–2.76, 9.63)	.28
	Overcoming barriers to exercise self-efficacy	–0.44 (–5.62, 4.74)	.87
	Exercise self-efficacy	0.12 (0.01, 0.23)	.03
	Exercise planning	0.18 (0.02, 0.34)	.03
**Stress and coping measures**			
	Symptoms of distress	0.08 (–0.01, 0.17)	.09
	Coping with stress	0.03 (–0.04, 0.10)	.37
	Aging beliefs	0.17 (0.04, 0.29)	.01

^a^ Adjusted for the corresponding baseline measure.

### Moderator and Dosage Effects Excluding Nonusers

Moderator analysis of the dataset excluding the 39 nonusers found that the interactions between gender × condition on overall exercise (*P=*.05), moderate exercise (*P=*.06), and sweat (*P=*.07) did not meet the threshold for statistical significance. As shown in Table 4, analysis of program effects conducted separately for males and females found significant program effects for females on overall exercise and exercise planning, with improvements in the desired direction on moderate exercise. No program effects were found for males on any of the exercise outcomes.

Multiple regression analysis of the dataset excluding the 39 nonusers examined the dosage effect of the number of pages viewed on all outcome variables. There were no significant associations between the number of pages viewed and any of the outcome measures after adjusting for the corresponding baseline measures.

**Table 4:** Adjusted program effects on dependent measures comparing program users and controls by gender.

Measure		Estimated adjusted posttest difference between program and control groups,^a^ Δ (95% CI)	*P*
**Godin: Moderate exercise**			
	Male	–0.06 (–0.71, 0.59)	.85
	Female	1.09 (–0.08, 2.27)	.07
**Godin: Sweat**			
	Male	0.15 (–0.06, 0.37)	.16
	Female	–0.20 (–0.53, 0.12)	.22
**Godin: Overall exercise**			
	Male	–0.37 (–7.63, 6.88)	.92
	Female	12.47 (1.20, 23.75)	.03
**Exercise planning**			
	Male	0.09 (–0.10, 0.27)	.34
	Female	0.39 (0.06, 0.72)	.02

^a^ Adjusted for the corresponding baseline measure.

## Discussion

### Principal Results

This randomized trial showed that working adults 50 years of age and older who were given access to the Web-based HealthyPast50 program showed significantly greater improvement on key health constructs over the 3-month test period than their 50 years and older counterparts in the control group. In the analysis of the imputed dataset, the program group performed significantly better than the control group on diet behavioral change self-efficacy, planning healthy eating, and mild exercise, and there were improvements on eating practices, moderate exercise, and overall exercise, but these did not meet the threshold for statistical significance. Moderator and dosage analyses of the imputed dataset found no significant effects. No significant program effects were found on measures of stress or aging beliefs. These results, following intent-to-treat principles and using multiple imputation methods, stand as the primary findings of the study and suggest that the HealthyPast50 program moved participants toward healthier eating and exercise practices. However, the program effects were selective because several measures of dietary and exercise outcomes showed no effects on participants.

Although the results from the analysis of the imputed dataset provide the most rigorous assessment of the program’s efficacy, the results of the parallel analyses on the nonimputed dataset comparing program users with controls are also of interest because they indicate the extent to which HealthyPast50 improved health outcomes for the participants defined as program users—those who used the program for at least 30 minutes (71.7% of the program group). These analyses found additional significant program effects on eating practices, exercise self-efficacy, exercise planning, and aging beliefs. Interestingly, the moderator analyses of the program users showed that program effects on exercise were due largely to significant effects on females, not males. An examination of pretest and posttest means on exercise measures indicated that the lack of significant improvement by men was at least partly a function of the fact that men in both groups exhibited much higher mean scores than women at pretest.

The effects of the Web-based program on multiple measures of diet and exercise are noteworthy, as numerous studies have shown diet and exercise to be critical behaviors—perhaps the most critical health behaviors after tobacco use—in reducing the risk of major disease. By reducing, at least partially, these key modifiable health risks in midlife, working adults might be laying the foundation for improved health and vitality in their later years.

These findings indicate not only that HealthyPast50 was more effective for women than men, but also that the program did not attract as many men with poor health practices as desired. There has not been a focus on these particular types of gender effects in studies of Web-based interventions; however, the role of self-efficacy and planning among women in this trial has a parallel in a study of an in-person exercise intervention tested in Finland [37]. At 3 months, increases in self-efficacy and planning among women predicted increases in their exercise levels; the same relationship was not found among men.

### Comparison With Prior Work

These findings are congruent in many respects to the findings from previous research by our group, as well as research by other investigators who have tested the efficacy of Internet-based interventions on the health practices of working adults. Across several randomized trials testing the effects of multiple Web-based programs on working adults, our group found significant effects on outcome measures of diet, exercise, stress, and substance misuse [17,19,22,38]. However, in this trial the effects of the HealthyPast50 program on dietary and exercise practices were somewhat stronger than in the trials of the other programs. The test of the Web-based Health Connection found effects on working adults’ dietary attitudes, but not on dietary or exercise practices [22]. Similarly, the Web-based Heart Healthy program exhibited significant impact on dietary attitudes and self-efficacy, as well as exercise practices, but did not show significant effects on eating practices [38]. Like Health Connection and Heart Healthy, HealthyPast50 is a multimedia, interactive, theory-based program. The main difference between HealthyPast50 and the previously tested programs is that the content of HealthyPast50 was tailored to—and tested on—adults aged 50 years and older.

To our knowledge, HealthyPast50 is the only Web-based program specifically developed for—and shown to be efficacious with—working adults 50 years and older. A recent trial of a health promotion program targeting older workers compared an in-person program with a publicly available Web-based program and found significant effects on both diet and exercise behaviors for the in-person program, but few effects for the Web-based program [11].

Tests by other investigators of Web-based programs only have found results similar to the findings of our trial. The multiple trials of the Web-based Guide to Health (GTH) program have generated impressive results on both dietary and physical behaviors [15,18]. Of particular interest is their study involving older participants (mean age 58.11 years) which showed significant effects on physical activity and related social cognitive theory (SCT) constructs [18]. In a causal model, increases in self-efficacy at 7 months led to increased physical activity levels at 16 months, suggesting that interventions with aging adults that boost self-efficacy might help older participants become more active [18]. These findings would seem to lend support to the view that the self-efficacy increases found in the HealthyPast50 trial contributed to the improvements in dietary and exercise practices, especially among women. In their randomized trial of a Web-based program to promote physical activity, Carr and associates found that compared to a control group with access to public health websites (eg, Mayo Clinic), the program group showed significant improvements at 3 months in total minutes of physical exercise, although the difference was not maintained at 6 months [16]. They attributed the promising effects of their Web-based program to the use of formative focus groups and the targeted inclusion of SCT elements in their program. With their Web-based programs rooted in SCT and containing multiple elements designed to increase self-efficacy and related SCT constructs, these interventions appear to be quite similar to HealthyPast50—and have generated similar positive effects on health behaviors.

### Limitations

Although this study exhibited a variety of strengths, including a randomized design, an advanced Web-based intervention, and a sizable workforce sample, the study also has some limitations, including—and perhaps foremost—the single posttest at 3 months and the reliance on self-reports. In addition, because of the particular characteristics of the sample, caution should be exercised in generalizing these findings to workforces that are less educated and affluent. Future research on HealthyPast50 should include longer-term posttests and the inclusion of physical measures (eg, weight, waist circumference, blood pressure). The program also needs to be tested on older workers who are less educated and affluent.

### Conclusions

The Web-based HealthyPast50 program demonstrated significant effects on the short-term dietary and exercise practices of older working adults. Significant program effects were not shown on measures of stress or aging beliefs, and there were too few smokers in the sample for meaningful analysis. Analysis of the nonimputed data indicated that program effects were stronger for women than men. The findings suggest that a multimedia Web-based program could be a promising vehicle for delivering health promotion material to older working adults.

## Multimedia Appendix 1

Trial protocol.

## Multimedia Appendix 2

Informed consent document.

## Multimedia Appendix 3

Selected screenshots from HealthyPast50.

## Multimedia Appendix 4

CONSORT-EHEALTH checklist V1.6.2 [39].

## Abbreviations

BMI: body mass index
GTH: Guide to Health
MCMC: Markov chain Monte Carlo
RCT: randomized controlled trial
SCT: social cognitive theory
