# Supplementary material for: A Web-Based Health Promotion Program for Older Workers: Randomized Controlled Trial
Source: J Med Internet Res. 2015 Mar 25;17(3):e82. doi: 10.2196/jmir.3399 (PMC4390614; doi:10.2196/jmir.3399)
Supplement: Supplementary file 1 [file jmir_v17i3e82_app1.pdf]

PROTOCOL SUMMARY FOR NEW AND CONTINUING PROTOCOLS

1**.** Provide a brief (200-250 word) summary of the background or statement of the problem.

As the American workforce ages, workplace-based risk reduction strategies that have been shown effective for the broad mainstream of workers offer particular promise for improving the health of older workers (ages 50 and older). With the aging of the baby boom generation, older workers comprise an expanding proportion of the workforce, now about one-third of the workforce, or approximately 50 million workers. Compared to younger workers, this group is at relatively high risk of chronic, debilitating diseases and costly health care services (NRC, 2004, Vita et al., 1998). Moreover, a significant percentage of these chronic diseases are preventable through the application of health promotion practices (Pelletier, 2005). Consequently, it seems likely that the application of health promotion interventions to this older group of workers can help delay the onset of aging and chronic disease, achieving health improvements and cost reductions in this segment of the population. In recent years, computer-based approaches to workplace health promotion and disease prevention strategies have become more numerous, and research has shown that tailored, media-rich web-based programs can improve worker health (Wantland et al., 2004; Rothert et al., 2006; Cook et al., 2007; Billings et al., 2008).

2**.** State purpose of study:

The overall goal of this project is to develop and test a comprehensive, interactive, and innovative web-based program designed to improve the health of older workers. This program will address a wide variety of health behavior topics, including physical activity, nutrition/weight management, stress management, mood management, alcohol use, and tobacco use – the health behaviors that contribute to the likelihood of contracting major diseases. The program will be tested with working adults 50 years of age and older.

3**.** Indicate the total number of subjects and the number of sites:

Number of subjects ___300______
Number of sites: _____1_______

4. Indicate the characteristics of study population:

(a) Gender: Males yes__X___ no______
Females yes__X__ no______
(b) Age range: from__50__ to ___80__
(c) Racial and Ethnic Groups:
Caucasian yes__X___ no______
Black yes__X___ no______
Hispanic yes__X___ no______
American Indian yes_____ no__X___
Alaskan Nativeyes__ _ __ no__X___
Asian/Pacific Islander yes__X___ no______
Other (specify) ______________
(d) Justify any exclusion of specific gender, age, and racial or ethnic groups:

The program specifically focuses on changing the health behaviors of people 50 years of age and as such, the 300 participants will be at least 50 years of age. The program is not designed for those under the age of 50.

We have not specifically excluded Alaskan Natives or American Indians. However, their base rates in the EMC employee population are quite low so we do not

anticipate that they will sign up for the study. Should any American Indian or Alaskan Native EMC employee choose to participate, they will be included in the study.

5. State inclusion criteria for enrollment in study:
All EMC employees 50 years of age and older will be recruited to participate in the field test of the employee program.

6. State exclusion criteria for enrollment in study:
The exclusion criteria will be age less than 50.

7. Will vulnerable subjects be enrolled in this study? yes_____ no___X___
   (a) Individuals with diminished mental capacity yes______ no___X___
   (b) children yes______ no___X___
   (c) pregnant women yes______ no___X___
   (d) fetuses yes______ no___X___
   (e) economically or educationally disadvantaged persons yes_____ no___X___
   (f) prisoners yes_____ no___X___

8. If vulnerable subjects are to be enrolled, describe the special precautions that will be taken to ensure that consent is freely given and that the rights and welfare of the subjects are protected:
N/A

9. If the study involves children, will a Certification of Assent form be used to document that assent was freely given without coercion? yes_____ no______
If no, indicate how assent will be documented:
N/A

10. Indicate where and how research data will be stored to ensure confidentiality:
The data will be obtained using a secure password protected online survey tool. The data will be stored in password protected computers and no names will be stored with the data.

Will data (e.g. records, samples, specimens, databases, surveys, etc.) be obtained with identifiers that can be directly or indirectly linked back to the subjects?
yes__X___ no______

There will be a separate file that links the Study ID to the individual. The study ID only, however, will be used in the database with the survey data.

12. Indicate who will have access to information about the subjects that is identifiable:

Member of the study team, not EMC, will have access to the data on a password-protected computer.

13. Indicate how potential subjects will be identified and recruited for participation in the study:
A flyer/email will be sent in a targeted email to all employees fifty years of age and older. EMC will send the flyer/email, not ISA. The flyer/email will indicate that the study is focusing on employees 50 years of age and older. Interested employees who fit the inclusion criteria (age 50 and older) will be asked to contact ISA at the study e-mail or telephone number. Even

though the flyer will be sent specifically to employees 50 years of age and older, the Project Manager will confirm the age at that time employees contact ISA.

When an employee contacts ISA, they will be provided with additional information about the project (see attached summary of what employees will be told when they contact ISA). The Project Manager will address any questions employees have and will then ask two questions to ensure that the employee qualifies for the study (age and whether they are an EMC employee).

14. Indicate when and where consent will be obtained:
    Consent will be obtained as part of the first survey data collection. It will be imbedded into the online survey and participants will not be able to continue with the survey until they have acknowledged and indicated that they consent.

15. Indicate how you will determine whether the subjects (or their surrogates) understand the information that was provided in the consent document:
    Participants will be asked to indicate on the consent document that they understood the information on the consent form. The telephone number for the project manager and IRB chair will be included if the participant has questions. A hard copy of the consent document will be e-mailed to participants.

16. Summarize, in a narrative what actually will be done to the subjects during their participation in the study. Make certain that the following are included:
    (a) a clear description of what is being done for research purposes and what is being done as part of standard clinical care;
    (b) a list of tests and procedures that will be performed for research purposes (e.g. blood tests, urine tests, cultures, interviews, questionnaires, surgical procedures, cardiac catheterization, pulmonary function tests, X-rays, scans, etc);
    (c) a brief description of the analyses that will be performed on the biologic or non-biologic (i.e. questionnaires) samples collected;
    (d) a list of investigational devices that will be used, indicate if they are classified as significant risk (SR) or non-significant risk (NSR) devices and whether there is an IDE or there is an application to the FDA for an IDE if the device is SR;
    (e) a statement that defines who will be financially responsible for the costs associated with participation in the study (e.g. examinations, procedures, drugs, devices, etc.) and a statement that defines what will be provided without cost to the subjects;
    (f) your assessment of whether the research involves any physical, psychological, social and/or economic risk(s) and the magnitude of the risk(s);
    (g) your assessment of the risk/benefit ratio of the research

NOTE:

a) Data and patient safety monitoring: if required, a Data and Safety Monitoring Board (DSMB), which must be convened by the PI, can be made up of internal and/or external members who have the appropriate expertise and are totally independent of and unaffiliated with the study. The composition of the DSMB should be commensurate with the complexity of the proposed study and will be reviewed by the IRB. Approval of the DSMB by the IRB is required prior to initiating the clinical trial.

Narrative:

(A) a clear description of what is being done for research purposes and what is being done as part of standard clinical care;

All EMC employees age 50 or older will be invited to participate in the field test of the program, approximately 300 of who are expected participate. ISA will then randomly assign 150 employees to the experimental condition and 150 employees to the control condition. Participation will be completely voluntary and employees will be recruited using a promotional campaign as well as monetary incentives ($25 per data collection) and cash drawing ($500).

All participants will be asked to complete the self-administered health questionnaire. The survey will be completed on-line through Survey Monkey. All data collected will be done for research purposes. Identification numbers will be used to link pretest and posttest surveys. Upon completion of the pretest data collection, participants will be randomly assigned to the experimental or control conditions. Participants in the experimental condition will be sent information on how to access the *HealthyPast50* program using a randomly generated unique identification number. Participants assigned to the program intervention will be encouraged to view the contents over multiple sessions during the three-month administration period. Employees will be encouraged to use the program during breaks and when not working. This method works best for facilitating optimal learning and helps to alleviate concerns that the worksite may have about time away from the job. Control group participants will be told that they will receive access to the *HealthPast50* program after the second survey. All participants will be allowed to access the EMC health programs as usual.

Approximately three months after experimental group participants receive initial access to the program they will be instructed that program viewing period is over. At that point all, participants will be sent information on how to complete the posttest questionnaire. That data collection will be conducted in a manner similar to the pretest, and as indicated above, once the posttest is complete, control group participants will be given access to the web-based program.

(B) a list of tests and procedures that will be performed for research purposes (e.g. blood tests, urine tests, cultures, interviews, questionnaires, surgical procedures, cardiac catheterization, pulmonary function tests, X-rays, scans, etc);

Outcome Measures

A draft copy of the survey questionnaire is attached. Most of the measures will be self-report scales of health behaviors that the ISA research team has used in several research projects during the past decade (e.g., Cook et al., 1996a, 1996b, 2004, 2005, 2007; Deitz et al., 2005; Billings et al., 2008)) and will therefore have the typically desired psychometric underpinnings -- established reliability coefficients, validity estimates, etc. -- as described below. In addition, several of the scales were developed and validated by other investigators – e.g., Block et al., 1986; Schwarzer and Renner, 2000; Glynn and Ruderman, 1986 – but have demonstrated evidence of reliability and validity. For all scales, however, psychometric analyses will be conducted on the measures once the data are in order to ensure that the collection of items can be legitimately summated; e.g., whether they have high Alpha coefficients and high item-total score correlations. The questionnaire will include the following measures:

- ❑ **Demographics --** Items assessing respondents' age, ethnicity, marital status, education, and income. These items have been associated with differences in health outcomes and will be

used as control variables in our multivariate models. They may also be used in interaction terms, or as moderators when applicable.

- **Symptoms of Distress** – A 15-item scale developed by Orioli et al. (1991) and used in multiple studies by our team (Cook, et al., 2003; Billings et al., 2008). Four items assessing the behavioral signs of stress (e.g., overeating, criticizing others) were summed with four other items assessing the physical signs of stress (e.g., muscle tension, headache). A recent randomized trial (Billings et al., 2008) generated an Alpha of .069. Evidence of validity was generated from Cook et al. (2003) and Billings et al. (2008) where the measure showed significant decreases in subjects exposed to stress management interventions.
- **Coping With Stress** – A 12-tems scale assessing the type of strategies one uses to cope with difficult situations and events. Two subscales are imbedded in the survey – adaptability and situation mastery. Adaptability measures flexibility in coping strategies and situation mastery measures the ability to recognize when and how to appropriately react to stressors. Questions are answered on a 4 point likert scale ranging from never to almost always. Typical questions include: "I often put things aside for a while to get perspective on them"; or "I decide certain problems are not worth worrying about". (Alpha for the scale was .76 in a previous ISA study).
- **Nutritional Patterns** – This 12-item scale assesses the nutritional value of the respondent's diet. It is a modification of the Block Self-Administered Diet History Questionnaire, developed and validated by Block and her associates (Block et al., 1986). The original Block questionnaire was found to correlate 0.70 with more detailed food recall methods, and a later validation study (Block et al., 1990) found that the questionnaire produced group mean nutrient estimates closely approximating the values obtained by three 4-day records. The Nutritional Patterns scale was used by our group in the test of the workplace "Healthy Eating" program with 210 employees of the GMAC insurance company (Cook et al., 2003), in which program participants showed significant improvements in their Nutritional Patterns scores from pretest to posttest ($F = 77.37$, $p < .001$) with an Alpha of 0.64.
- **Attitudes Toward a Healthy Diet** – This 17-item scale, based on the Health Belief Model, was developed and validated by Trenkner and associates (Trenkner et al., 1990), assesses perceived benefits and barriers to eating a healthy diet. Developed through a sequence of four samples, the administration of the final version to the last sample of 808 respondents yielded an Alpha of .87. The concurrent validity of the scale was demonstrated by significant associations between the scale and other measures of nutrition attitudes and eating behavior. This scale was used as an outcome measure in our test of the "Healthy Eating" program, with program participants showing significant improvements in their scores from pretest to posttest ($F = 41.04$, $p < .001$), and in our RCT of the "Health Connection" program, with program participants showing significant improvements in their scores from pretest to posttest ($t = 7.67$, $p < .001$)
- **Eating Practices** -- This 10-item subscale is part of the Weight Control Assessment scale developed by O'Neil & Rhodes (in Wolfe, B.L., 1996), and assesses the extent to which the respondent exercises control over their eating. It contains items such as, "How often do eat between meals," and "Do you have trouble controlling your eating when your favorite foods are around the house?" This subscale was also used in our Healthy Eating field test, with program participants showing significant improvements in their scores from pretest to posttest ($F = 20.22$, $p < .001$), with an Alpha of .63. It was also used in the RCT of our web-based "Health Connection" program (Cook et al., 2007) in which participants showed significant improvements in their Eating Practices scores from pretest to posttest ($t = 4.72$, $p < .001$).

- **Overeating Self-Efficacy Scale** – A 14-item scale assessing one's confidence in resisting overeating in different situations. Developed by McCann et al. (1995), this scale is a shortened version of the 25-item Eating Self-Efficacy Scale (Glynn & Ruderman, 1986). Glynn and Ruderman (1986) reported an Alpha of .92 and a test-retest reliability of .70 over a 7-week period (N= 484), along with evidence of convergent validity (e.g., ESES scores significantly correlated with dieting behavior).
- **Diet Behavioral Change Self-Efficacy** – A 5-item scale assessing perceived self-efficacy to engage in more healthful eating practices over the next month. Questions are answered on a 5 point Likert scale ranging from 'Not Confident' to 'Extremely Confident'. Typical questions on this scale include: "How confident are you that you have the skills to eat a healthy diet?"; Or "How confident are you that you have the skills to control the amount or portion size of food that you eat at each meal?". This survey has been used in other ISA studies (Alpha .83).
- **Height and Weight** – Two items will ask the respondent will be asked to report their height and weight. We do not anticipate checking the validity of that response with actually height and weight measurements.
- **Exercise Habits** – The Godin Leisure-Time Exercise Questionnaire (Godin and Shephard, 1997) is a brief four-item query of usual leisure-time exercise habits. The first three items ask the respondent to indicate the times per week they engage in strenuous, moderate, and light activities. The fourth item asks how often in a typical 7-day period the respondent engages in activity long enough to work up a sweat. Reliability and concurrent validity of the measure demonstrated by Godin and Shephard (1997). Also used in our RCT of the "Health Connection" (Cook et al., 2007).
- **Overcoming Barriers to Exercise Self-Efficacy and Self Efficacy to Engage in Regular Exercise** – Two separate measures will assess Self Efficacy for Overcoming Barriers to Exercise and Self Efficacy to Engage in Regular Exercise. The Overcoming Barriers Self Efficacy measure is a 13-item measure developed and validated by McAuley and colleagues (McAuley et al., 1990; McAuley, 1992) asking users to rate on a scale from 0-100 the percentage of confidence they have to exercise in a variety of situations that might be considered barriers to activity. Alpha for this measure was .88. The second measure, an 8-item scale assessing one's confidence in being able to engage in regular exercise. This measure was developed by Kroll et al. and is answered on a 4 point Likert scale ranging from 'Not at All True' to 'Always True'. Typical questions on this scale include: "I can overcome barriers and challenges with regard to physical activity and exercise if I try hard enough"; and "I can accomplish the physical activity and exercise goals that I set". (Alpha = .89).
- **Planning Physical Activity** – A 2-item measure adapted from Soureti, Schwarzer, and Renner assessing the extent to which the participant has a plan for engaging in physical activity.
- **Planning Healthy Eating** – Similar to the planning for physical activity, this 2-item measure is also adapted from Soureti, Schwarzer, and Renner and assesses the extent to which the participant has developed a plan for eating a healthy diet.
- **Tobacco Use** -- Originally used in a study of a workplace smoking cessation program (Jeffery et al., 1993) and adapted by our team in a test of a workplace substance abuse prevention program (Cook et al., 2004), will assess one's use of tobacco. Items include, 1) whether the person smokes cigarettes or uses other tobacco products, 2) the number of days in the past 30 days the person smoked a cigarette or used a tobacco product; 3) the typical number of cigarettes smoked or times tobacco used per day in the past 30 days, 4) whether the person has ever tried to quit, 5) whether the person would like to quit, and 6) number of quit attempts in the past year.

- **Alcohol use quantity/frequency*y*.** A widely used measure of alcohol consumption, consisting of four items (not a summated scale) that assess: 1) Whether the respondent had a drink in the past 30 days; (2) the number of days in the past 30 days the subject had a drink, 3) the number of drinks usually drunk on those days, and (4) the number of days the subject had five or more drinks at one time. Used in numerous studies of alcohol use in the workplace, including several of our studies (e.g., Cook et al., 1996b; Cook et al., 2004; Deitz et al., 2005).
- **Belief About Aging** – This 5-item measure, developed by the study team will assess participants beliefs about aging.

(C) a brief description of the analyses that will be performed on the biologic or non-biologic (i.e. questionnaires) samples collected

Analysis of primary outcomes.

The data will derive from a randomized controlled trial (RCT) with random assignment of individual participants to one of two study conditions (intervention vs. control). Data will be collected at baseline and posttest. The primary outcomes include measures of diet, weight, physical activity, stress, depression, alcohol use and tobacco use. Each of these measures will be self-reports using established scales that have evidence of reliability (internal consistency and/or test-retest reliability) and validity, as described below..

Primary analysis. The primary analysis will be an analysis of covariance (ANCOVA) wherein the intervention and control conditions are compared at posttest with regression adjustment for baseline values on the dependent variable. We will also consider using the measure of Health and Job Control as a covariate, along with potential confounders, as mentioned below. The test of the intervention effect will be the t-test for the regression coefficient for condition, which will estimate the adjusted difference between the two conditions at posttest. That test will be two-tailed with a type I error rate of 5%. We will perform the analysis using SAS PROC GLM, Version 8.2 (SAS Institute, 2001).

Assumptions. ANCOVA is based on the General Linear Model (Searle, 1971). It assumes that the dependent variable is a linear function of the predictors, that the errors are independent, and that the errors have a normal distribution. These assumptions are commonly met in most RCTs involving continuous dependent variables, especially when treatments are delivered to individuals, as will be the case here. Even so, we will examine the residuals from the models to confirm that they are normal. Our experience with these variables indicates that the assumption of normality will be met. If it is not, we will assume a more appropriate distribution or consider a transformation to normalize the data.

Missing Data. The primary analysis will employ intention-to-treat principles. Because randomization carries the expectation of creating treatment groups equivalent with respect to known and unknown prognostic factors, removing randomized participants from the analysis runs the risk of tampering with this balance and introducing bias into the treatment comparisons. As a result, all participants randomized into the study will be included in all analyses irrespective of protocol violations and events arising post randomization and all participants will be analyzed according to the treatment to which they were randomized. We will make a concerted effort to track, locate and measure all participants at the follow-up surveys. However, even with our best efforts, we can anticipate that there will be some missing data; based on our previous work, we estimate that we will lose no more than 20% of the sample. Multiple imputation is now widely regarded as an effective method for replacing missing data. In particular, we will fit a logistic regression equation using baseline data from participants in the control condition to predict participation status at follow-up. We will use that equation to generate a predicted value to replace each missing value in both conditions,

adding a value for residual error generated from a distribution matching the follow-up participation status distribution in the comparison condition. By repeating that procedure several times, we will obtain several complete data sets that differ only in the values imputed to replace the missing data. We will then analyze those data sets and combine their results to provide a summary estimate and test for the intervention effect. We will employ SAS PROC MI and SAS PROC MIANALYZE, Version 8.2, to implement these multiple imputation procedures (SAS Institute, 2001).

Confounding. Though randomization of 150 participants to each condition makes it unlikely that there will be any imbalance of prognostic factors between the two conditions, confounding remains a possibility in any single realization of an experiment. Left alone, such imbalance could confound the true relationship between condition and the outcomes. In order to avoid this problem, we will examine prognostic factors measured at baseline for evidence of any imbalance; if it is present, we will perform secondary analyses in which we repeat the primary analysis adding the potential confounders as additional covariates in the ANCOVA, attending carefully to issues of measurement error, which can create problems for such adjustments. Regression adjustment cannot completely correct for confounding, but to the extent that the confounders are well measured and properly modeled, the adjusted analysis will reduce the bias created by those confounders.

Effect modification. Other secondary analyses will explore the assumption of homogeneity of the intervention effect across subgroups of participants. Though power will be limited for interactions, we will add a subgroup main effect and a condition x subgroup interaction to the ANCOVA in order to explore possible interactions. The primary subgroup variables will be gender, age group, and racial or ethnic group.

Alternative analytic strategies. Several alternative analytic strategies were considered. The data could be analyzed using a repeated measures analysis of variance model, wherein condition, time (baseline and the two posttests) and their interaction are included as independent variables. However, this approach tends to have less power than the analysis proposed here, and it also makes unnecessary assumptions about the structural relationship between the baseline and posttest values of the outcome variables. For those reasons, we prefer the ANCOVA.

(D) a list of investigational devices that will be used, indicate if they are classified as significant risk (SR) or non-significant risk (NSR) devices and whether there is an IDE or there is an application to the FDA for an IDE if the device is SR;

N/A

(E) a statement that defines who will be financially responsible for the costs associated with participation in the study (e.g. examinations, procedures, drugs, devices, etc.) and a statement that defines what will be provided without cost to the subjects;

ISA will be responsible for all costs associated with participation in the study. Participants will be asked to complete the survey and program review (for experimental group participants and managers) on their own time and not during work time.

(F) your assessment of whether the research involves any physical, psychological, social and/or economic risk(s) and the magnitude of the risk(s);

The potential risks of collecting the data are quite minimal. It is possible that participants could become uncomfortable when responding to the survey questions. To minimize this risk, participants will complete their questionnaires in private and their names will not be associated with their data. In addition, the survey questions will be phrased in ways to minimize any

potential discomfort. While the risks of the field survey are minimal, it is possible that a participant may, as a function of participating in the field test, recognize one or more serious medical problems requiring immediate attention. The program will include a number of resources that the participant can use and steps to take in the event of an acute or serious problem. Users will be referred to the resources section at several junctions of the program and a list of resources both nationally and locally, will be provided to those requiring further assistance. A disclaimer will be listed at the beginning of the program that says the site is designed to educate users and provide them with the skills to effectively reduce their risk for disease, but that they should seek the advice of a medical care provider if additional assistance is needed. Additionally, the consent form will point out this potential risk and inform participants that they should call their health care provider if they need immediate assistance.

(G) your assessment of the risk/benefit ratio of the research;

The potential risks of the research to participants seem minimal, and are far outweighed by the anticipated benefit. These benefits include the development of an effective, interactive web-based program that can educate older workers on health risks and wellness and prevent health and financial problems associated lifestyle factors. All participants will benefit by receiving access to the *HealthyPast50* program (either as part of the program evaluation or, for control group participants, after the surveys are completed). We believe that the minimal risk associated with participation is quite reasonable when compared to the benefit that participants and the larger society can receive from reducing or preventing the development of acute or chronic disease among disease among older workers.
