# Supplementary material for: A Web-Based Health Promotion Program for Older Workers: Randomized Controlled Trial
Source: J Med Internet Res. 2015 Mar 25;17(3):e82. doi: 10.2196/jmir.3399 (PMC4390614; doi:10.2196/jmir.3399)
Supplement: Supplementary file 2 [file jmir_v17i3e82_app2.pdf]

Consent Form

WEB-BASED HEALTH PROMOTION FOR WORKING ADULTS AGE 50 AND OLDER

Nature and Purpose of Study

ISA Associates, a research firm located in Alexandria, Virginia, has received a grant from the National Institutes of Health to develop and test a web-based health promotion program for workers aged 50 and older. ISA is working with EMC to provide this new program to employees 50 years of age and older. The goal of this program is to inform users about health risks common to people 50 years of age and older and ways to reduce risk. The program provides information and educational materials on healthy eating, exercise, reduction in personal stresses, how to stop smoking, and specific recommendations for reducing health risks among other health topics. As part of the study, we would like you to complete a confidential questionnaire (on a total of two occasions) and use the web-based program that will be provided to you. You are being asked to participate in a research study to test the effectiveness of this program.

Explanation of Procedures

As part of the study, we would like you to complete a confidential health questionnaire now and again in 3 months. Completion of each questionnaire will take 30-45 minutes. The questionnaire asks about general health practices (i.e. diet and exercise), stress and coping, height and weight, confidence for engaging in healthful practices, and tobacco use. The questionnaires are completed on-line through a secure web-based system. After completing the first questionnaire, half of those in the study will be selected at random to use the new web-based program immediately. The other half will receive the health and wellness services they would normally receive and will be able to use the new program after the second questionnaire.

Participants randomly selected to use the program between the first and second questionnaire will be asked to use the program at their own pace over a period of three months. We suggest that participants log into the program each week, perhaps for an hour, and review those sections most relevant to them. To assist you throughout the program review process, you will receive periodic reminders about using the program.

You will be assigned a unique identification number that you can use to log into the on-line questionnaire. We WILL NOT use your name in any database that includes your questionnaire responses or report your responses to your employer or anyone outside the ISA research team. Your responses will assist us in testing the effectiveness of this new program. Please remember your responses are completely confidential.

Payment for Participation

You will receive $25 for completing the first questionnaire and $25 for completing the second questionnaire. In addition, your name will be entered into a drawing where one participant will receive $500 during each questionnaire round.

Additional Costs

There are no additional costs for participating in this project.

RISKS AND DISCOMFORTS

You may experience discomfort in reviewing the program and completing some of the questions on the questionnaire, particularly if by doing so you recognize some health risks or problems that you were not aware of, but we believe that this discomfort will be minimal. Some of the items on the questionnaire might make you feel uncomfortable because they are personal. You do not have to answer any question that makes you feel uncomfortable. Please remember, that all of your responses will be kept completely confidential. As detailed below in the "Confidentiality" section, each participant will receive a unique study identification number. Your name will not be on any of the questionnaires you complete. In addition, all research findings will be summarized in group form only.

You may, as a result of reviewing the program, have concerns about your mental or physical health. If you have ANY concerns about your health, we recommend that you seek the immediate assistance of a medical or behavioral health care provider such as your physician, family care provider, or an emergency service provider. You may also contact the Employee Assistance Program at 866-808-5062. If you need help finding a health care provider, please contact Dr. Rebekah Hersch, ISA Project Director at 800-662-9976 Ext. 11.

POSSIBLE BENEFITS

Beyond payment for participation, we believe that you will learn valuable information about how to engage in healthy lifestyle practices. The information we learn from you will help us design a state-of-the-art web-based program to help individuals 50 years of age and older modify/reduce health risks and prevent the development of medical problems.

CONFIDENTIALITY

All data collected online will be done through a secure website. You will use a unique identification number to enter the web-based program and complete the online questionnaires. Your name and contact information will be kept separately from all your other data. You will not enter any personally identifying information when using the program or completing a questionnaire. None of your responses to the questionnaire will be shared with your employer.

OTHER PEOPLE TO WHOM QUESTIONS CAN BE ADDRESSED

If you have additional questions, you may contact the Project Manager, Rebekah Hersch, Ph.D. toll-free at 800-662-9976 ext 11. You may also obtain additional information about your rights as a research subject from Diane Deitz, Ph.D., Institutional Review Board, toll-free at 800-662-9976, ext. 17. If you have a question or feel uncomfortable about your participation in this study, these are the individuals you should contact between the hours of 9:00-5:00 EST, Monday through Friday.

EXPLANATION OF ABILITY TO WITHDRAW FROM STUDY

Your participation is completely voluntary. You may discontinue participation at any time. You may also skip any question on the questionnaire. If you decide not to participate or to discontinue participation, this will not affect your employment in any way.

Consent to Participate

I understand that this study will be supervised by Rebekah Hersch, Ph.D. and whomever she may designate as her assistants. I have read this explanation of activities to be followed. With this knowledge of the nature and purposes of the activities, the possible risks and discomforts, and the possible benefits, I hereby authorize the performance of the activities described above. I willingly consent to be a part of this research study. I understand that I will receive a copy of this Consent Form by email.
