# Supplementary material for: A Web-Based Health Promotion Program for Older Workers: Randomized Controlled Trial
Source: J Med Internet Res. 2015 Mar 25;17(3):e82. doi: 10.2196/jmir.3399 (PMC4390614; doi:10.2196/jmir.3399)
Supplement: Supplementary file 3 [file jmir_v17i3e82_app3.pdf]

Build Your Active Lifestyle

Planning ahead is an important part of getting and staying active. Taking the time to plan your activities in advance will not only make it easier to commit to being active, but will help you build a balanced program that trains each component of fitness at the recommended levels.

Use the two-week calendar below to begin building your active lifestyle. For each day in the calendar, you can specify what types of activities you will do at what times and for how long. You'll notice that the calendar has room for up to four activities, but this doesn't mean you need to plan four different activities each day! Use the **sample schedule** as a guide. Keep in mind, though, the sample schedule is just to give you an idea of how to use this calendar. Plan your activity so that it fits with your schedule, likes and dislikes, and personal goals. Strive to achieve at least the minimum recommendations for each of the components of fitness. To track your progress, print your calendar and check off your activities as you complete them.

- ***Click on any of the four components of fitness icons below for example activities and minimum training recommendations.***
- ***Click on any square in the calendar below to add an activity to your schedule. Once you've added an activity you can click on the square again to see the details. You can also click the Print Schedule button for a PDF version of your entire calendar that you can print or save.***

CARDIOVASCULAR FITNESS | MUSCULAR STRENGTH AND ENDURANCE | FLEXIBILITY | NEUROMOTOR FITNESS

Clear Schedule | Print Schedule

Week 1

MONDAY	TUESDAY	WEDNESDAY	THURSDAY	FRIDAY	SATURDAY	SUNDAY

Download:

Sample Schedule (PDF)

Related Topics:

Balanced Fitness

How Much, How Hard, How Often?

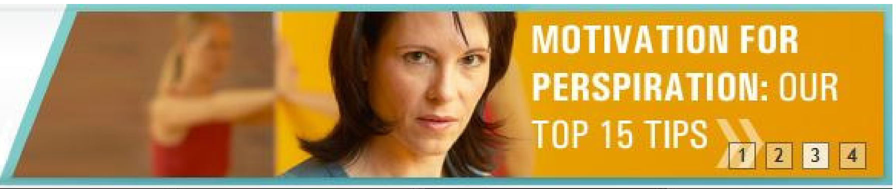

Active Lifestyle

Living an active lifestyle is a key component to health and longevity. And it's never too late to begin. No matter what your prior experience is with physical activity, **this module will provide you with the knowledge and motivational tools to start or continue being physically active.**

If this is your first time visiting the Active Lifestyle module (or you haven't been here in a while), we suggest starting with the **How Active Are You Really?** physical activity assessment. After completing the assessment, you'll receive feedback guiding you to the pages that may be especially relevant to you.

Then, feel free to browse the entire module. You'll find a wealth of up-to-date, scientifically based information on starting and staying active, as well as strategies to make it all work for you!

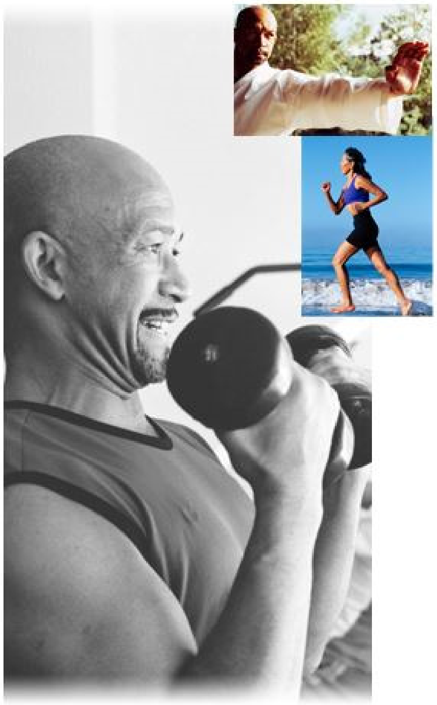

When you're ready to begin, click on any of the topics listed below.

ACTIVE LIFESTYLE ASSESSMENTS

How Active Are You, Really?

Physical Activity Readiness Questionnaire (PAR-Q)

WHY BE PHYSICALLY ACTIVE?

The Body of Evidence

What is Fit?

STARTING AND STAYING ACTIVE

What's Holding You Back?

Setting S.M.A.R.T. Goals

Top 15 Tips for Staying Motivated

Balanced Fitness

How Much, How Hard, How Often

Measuring Intensity

Adjusting Intensity

Structuring Your Physical Activity Session

Fueling Your Activity

Preventing Injury and Illness

Testimonials

I feel better than I did at 43...

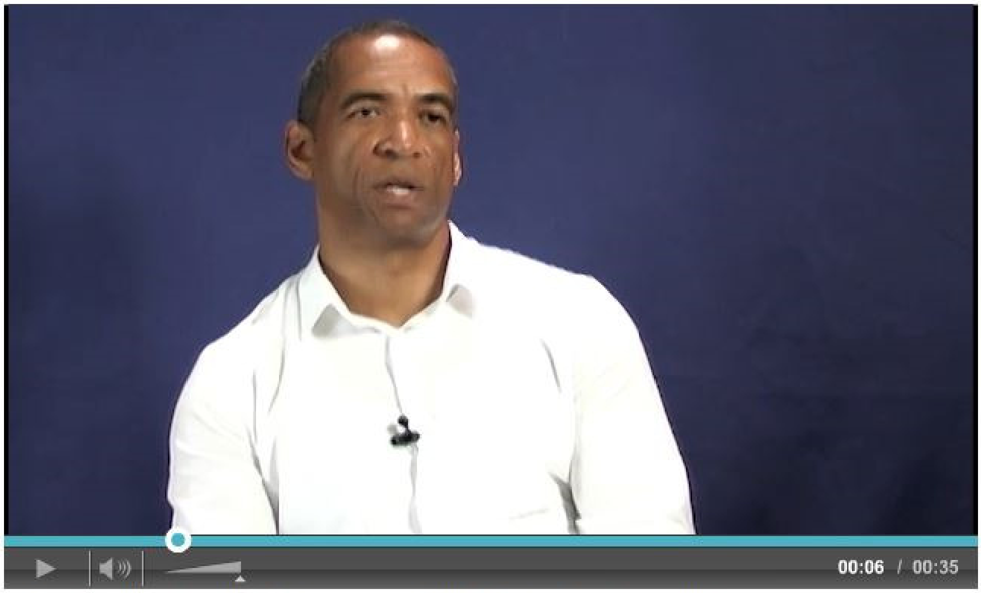

00:06 / 00:35

Living a healthy, active lifestyle has Donnie feeling better today than he did 10 years ago.

regular physical activity can elminate aches and pains.

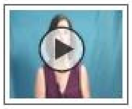

Walking is always something you can do.

You don't need to go to a gym to be physically active. Listen to Sally's ideas.

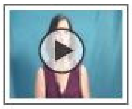

Get your friends to do it with you.

Sally discusses how social support can help you become more physically active.

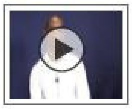

I feel better than I did at 43...

Living a healthy, active lifestyle has Donnie feeling better today than he did 10 years ago.

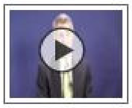

The more I ran, the better I felt.

Scott describes how he became more physically active- and [illegible]

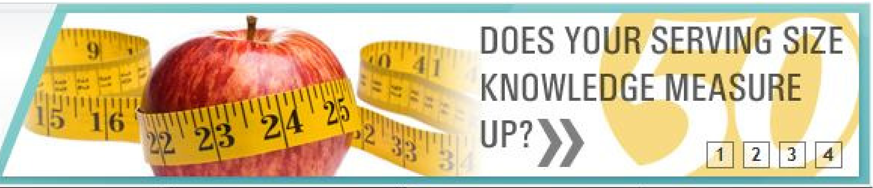

Healthy Eating

We've known for some time that our daily food choices impact our health and well-being. Yet the rates of overweight, obesity, and chronic illnesses continue to climb! To make matters more complicated, our nutritional requirements change as we age. Add in ever-increasing portion sizes, fad diets, and evolving nutrition research, and *figuring out what to eat can be truly confusing!*

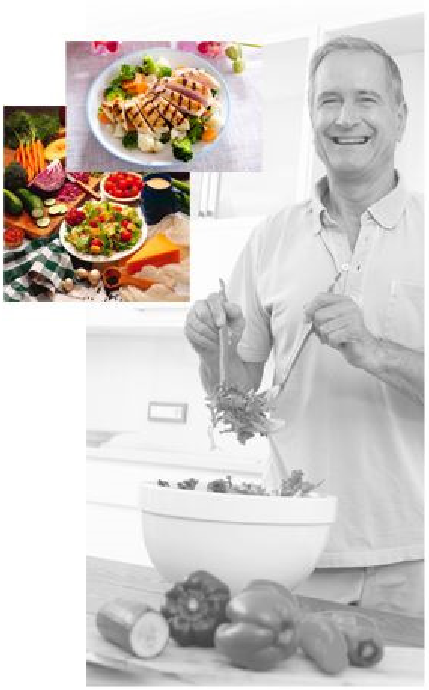

The Healthy Eating module is designed clear up the confusion—and to provide you with the tools and skills you need to *make lasting changes to your diet*. This module is designed for everyone, regardless of your nutritional goals. While it is not a weight loss program, per se, many people have lost weight using the strategies in this program.

If this is your first time visiting the Healthy Eating module (or you haven't been here in a while), we suggest starting with the **My Nutritional Patterns** assessment. After completing the assessment, you'll receive feedback guiding you to the pages that may be especially relevant to you. Then, feel free to browse the entire module. You'll find a wealth of up-to-date, scientifically based information on nutrition, weight management, as well as strategies to make it all work for you!

When you're ready to begin, click on any of the topics listed below.

HEALTHY EATING ASSESSMENT
My Nutritional Patterns

WHY BE FOOD SMART?
The Body of Evidence

WEIGHT MANAGEMENT
A Balanced Approach to Weight Management
Meeting Challenges
Refusal Skills
Secrets of Satiation
De-Mystifying Diets

STRATEGIES FOR HEALTHY EATING
Mindful Eating
Understanding Food Labels
Shop Smart
Smart Cooking
Healthy Dining Out
Practice Portion Control
Food Smart Substitutions

Practice Portion Control

When it comes to achieving or maintaining a healthy weight, practicing portion control is the key to success! Since portion sizes have been growing steadily in North America over the past 30 years, it can be very difficult to figure out what an acceptable portion really looks like. Take this quiz to see how well you know what different serving sizes really look like.

For each question, after you've selected an answer, click the "Submit" button for feedback.

1/7 A half cup serving of cooked pasta is about the size of...

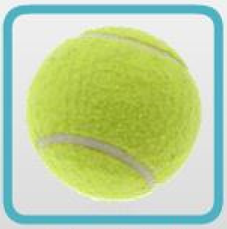

A tennis ball

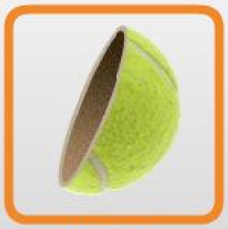

Half of a tennis ball

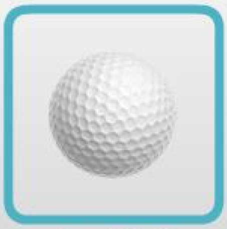

A golf ball

Select an answer by clicking on an image.
Then click the Submit button to confirm your choice.

Submit

Use these buttons to page through this Module.

Previous | Next

Go to Module Menu Page

Testimonial:

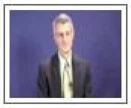

I don't feel the need to clean my plate.

Listen to Scott talk about how he controls his portion sizes when dining out.

Additional Testimonials

From the Toolbox:

Serving Size Reference Guide

Related Topics:

Mindful Eating

Understanding Food Labels

HealthyPast 50
Your Guide to Feeling Young & Vital

Welcome to Healthy Past 50!

If this is your first time visiting the site, we recommend starting with **Facts About Healthy Aging**. Then assess your current health status with the **My Health Profile Assessment**.

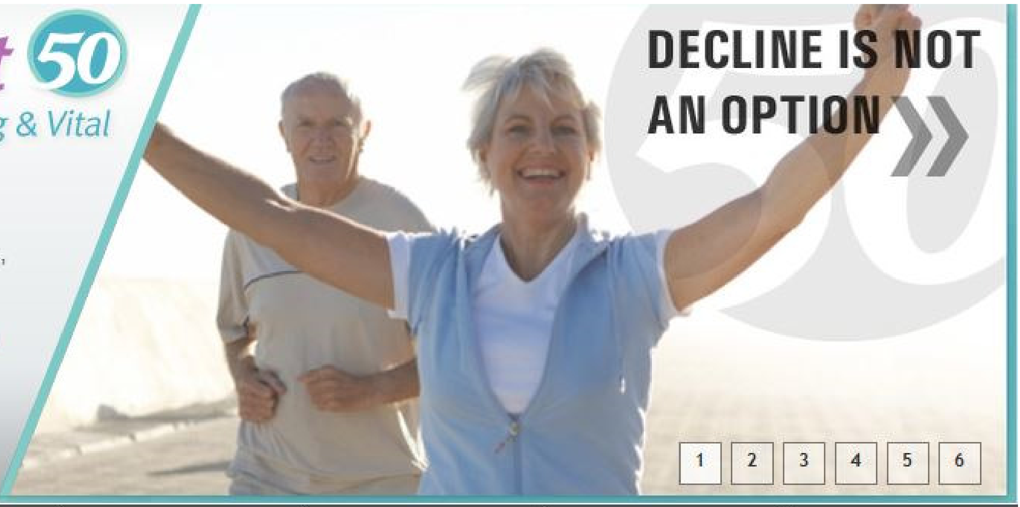

NEW MATERIAL: Click on the links below to learn how exercise and meditation can boost your immune system and help you fight off colds and the flu.

Exercise to help boost your immune system

Exercise and meditation can fight colds and flu

About Healthy Past 50

- *Click on the video below to learn how Healthy Past 50 was developed and how it differs from other websites. Click here to listen to the developers describe their vision for Healthy Past 50.*

Stress and Age

Stressors and how we respond to them change as we age. The things that may have caused great stress in our 20s and 30s are often very different than the events and circumstances that cause stress in our 50s and 60s. The key is knowing what your stressors are and how to manage them.

In **Stress and Your Body**, we described how acute and chronic stress impact the body. While we know that stress impacts everyone differently, one thing is clear, chronic perceived stress negatively impacts the body at the *cellular* level. To explain what we mean, let's take a look at stress and the aging process.

Play the video below to learn about the relationship between stress and aging.

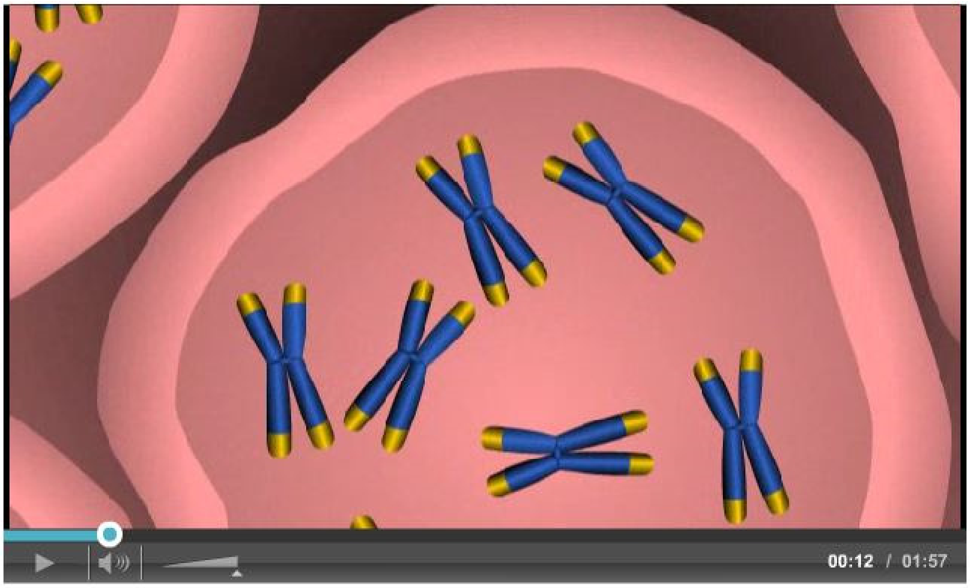

Use these buttons to page through this Module.

Previous | Next

Go to Module Menu Page

Testimonial:

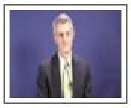

There's a benefit to being a certain age... it's easier to address problems.

For Scott, managing stress has gotten easier with age.

Additional Testimonials

Related Topics:

Good and Bad Stress

Stress and Your Body

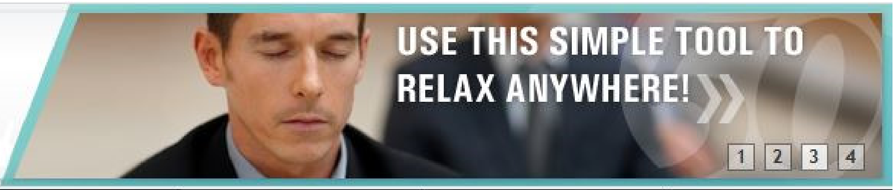

Stress & Mood Management

Stress is a normal part of our daily lives. It can motivate us to achieve our goals, or it can cause us to feel overwhelmed. How we view and deal with stressors is important to our overall health and well-being. When stress exceeds our ability to cope, it puts us at risk for a range of physical and mental health challenges. In fact, chronic stress actually accelerates the aging process. So, while our stressors may change as we age, it's never too late to adopt proven strategies to manage stress.

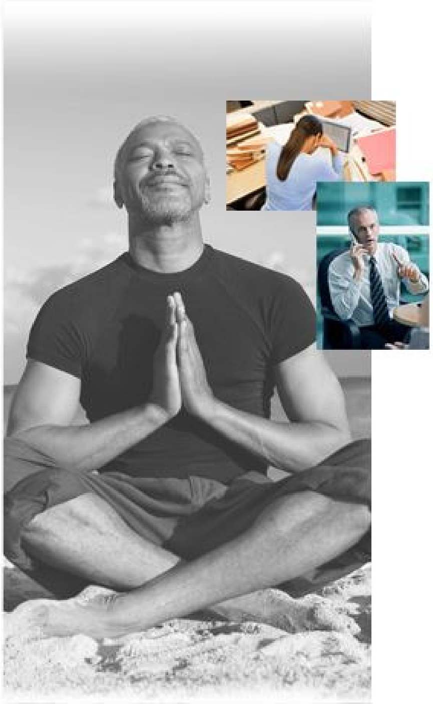

The Stress & Mood Management module will help you understand what stress is and how your personal stressors are affecting your health. The *Manage Stress* section is really the heart of this module. In it, you'll find a wide variety of powerful techniques, many of them interactive and downloadable, to address your personal stressors. In this module, we'll also touch on how to recognize and get help for depression and anxiety, two common mental health disorders that can result from chronic stress.

When you're ready to begin, click on any of the topics listed below.

ASSESS YOUR STRESS

What's My Stress Level?

What's My Coping Level?

My Stress Profile

THE IMPORTANCE OF MANAGING STRESS

Good and Bad Stress

Stress and Your Body

Stress and Age

MANAGE STRESS

The Basics of Stress Management

Reacting vs. Responding

Change Your View of Stressors

Mental Lens

Learned Optimism/Authentic Happiness

Building Resilience

Mindfulness

Changing Negative Thinking

Change Your Body's Response to Stress

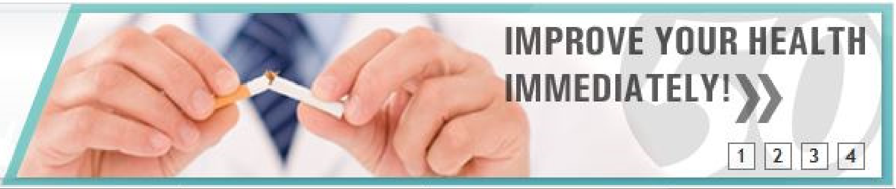

Tobacco Free

If you're a smoker, chances are you know you need to quit. You may not WANT to quit. You may have some seemingly good reasons for putting it off. But the truth is, quitting smoking is perhaps the single most important thing you can do for your health.

If you're willing to try, this module can empower you to make a decision to quit—once and for all—and give you the strategies you need to make it work.

You can go through this module in any order. However, if this is your first time visiting (or you haven't been here in a while) we suggest you begin with **Assess Your Tobacco Use**, where you'll receive feedback guiding you to the pages that may be especially relevant to you. Of course, we encourage you to view the entire module where you'll have a chance to look objectively at how you use tobacco, learn about the benefits of kicking your habit, and discover some new tricks and strategies for giving up tobacco for good!

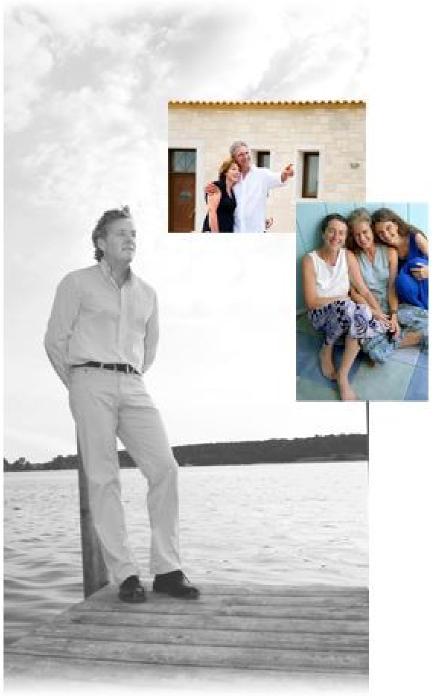

When you're ready to begin, click on any of the topics listed below.

ASSESS YOUR TOBACCO USE
Are You Dependent?

WHY BE TOBACCO FREE?
The Body of Evidence
Nicotine is a Drug
Improve Your Health Immediately
What Doctors Say

PREPARING TO QUIT
Understanding My Smoking Habit
Setting S.M.A.R.T. Goals
Plan Your Quit Program
Preparation Checklist
Getting Through Quit Day

REMAINING TOBACCO FREE
Healthy Alternatives
The Importance of Social Support
Learning to Say "NO!"
Managing Triggers
