# Supplementary material for: A Web-Based Health Promotion Program for Older Workers: Randomized Controlled Trial
Source: J Med Internet Res. 2015 Mar 25;17(3):e82. doi: 10.2196/jmir.3399 (PMC4390614; doi:10.2196/jmir.3399)
Supplement: Supplementary file 4 [file jmir_v17i3e82_app4.pdf]

CONSORT-EHEALTH Checklist V1.6.2 Report

(based on CONSORT-EHEALTH V1.6), available at [http://tinyurl.com/consort-ehealth-v1-6].

3399

Date completed

3/12/2014 13:15:11

by

Royer Cook

A Randomized Controlled Trial of a Web-Based Health Promotion Program for Older Workers

TITLE

1a-i) Identify the mode of delivery in the title

"Web-Based Health Promotion Program for Older Workers"

1a-ii) Non-web-based components or important co-interventions in title

There were no non-web components.

1a-iii) Primary condition or target group in the title

..."Program for Older Workers."

ABSTRACT

1b-i) Key features/functionalities/components of the intervention and comparator in the METHODS section of the ABSTRACT

"HealthyPast50, an automated web-based health promotion program based on social-cognitive theory....
... randomly assigned to the web-based HealthyPast50 program or to a wait-list control condition."

1b-ii) Level of human involvement in the METHODS section of the ABSTRACT

"...HealthyPast50, a fully automated web-based health promotion program..."

1b-iii) Open vs. closed, web-based (self-assessment) vs. face-to-face assessments in the METHODS section of the ABSTRACT

"The sample included 278 employees aged 50 to 68 who were recruited online and randomly assigned to the web-based HealthyPast50 program or to a wait-list control condition. Self-report measures of diet, physical activity, stress, and tobacco use were collected online before and three months after the program group was given access to the program."

1b-iv) RESULTS section in abstract must contain use data

"Retention rates were good for both groups – 80% for the program group and 94% for the control group. Program group participants logged in an average of 2.15 times (SD = 3.71) and viewed an average of 11.04 pages (SD = 20.08)."

1b-v) CONCLUSIONS/DISCUSSION in abstract for negative trials

N/A - trial was not negative.

INTRODUCTION

2a-i) Problem and the type of system/solution

"This program, called HealthyPast50, was designed as a stand-alone intervention to address a wide variety of health behavior topics, including physical activity, healthy eating, stress management, and tobacco cessation – the health behaviors that contribute to the prevention of major diseases."

2a-ii) Scientific background, rationale: What is known about the (type of) system

"Research on web-based programs indicates that such approaches can be an effective means of contributing to positive changes in diet, physical activity, stress management and substance misuse [14-19]. However, with the exception of the study by Hughes and associates [11], none of the more promising studies focused on older working adults..."

METHODS

3a) CONSORT: Description of trial design (such as parallel, factorial) including allocation ratio

"The purpose of the research was to evaluate the impact of a multi-media web-based health promotion program on central health practices of older workers."

3b) CONSORT: Important changes to methods after trial commencement (such as eligibility criteria), with reasons

"A "project update" email was sent to participants at one-month and two-months post randomization. For the program group, the emails included a reminder to use the program and information about the latest update to the program (a brief message on the home page). With the exception of the project update, the program was unchanged throughout the test period."

3b-i) Bug fixes, Downtimes, Content Changes

"With the exception of the project update, the program was unchanged throughout the test period."

4a) CONSORT: Eligibility criteria for participants

"...all employees 50 years of age and older (approximately 2,500 employees) located in multiple U.S. offices of a global information technology company."

4a-i) Computer / Internet literacy

Because the sample consisted of workers in a global technology company, "The computer literacy of the workforce was estimated to be relatively high...

4a-ii) Open vs. closed, web-based vs. face-to-face assessments:

"A recruitment flyer briefly describing the purpose of the study was emailed by company officials to all employees 50 years of age and older (approximately 2,500 employees) located in multiple U.S. offices of a global information technology company." Identification was quasi-anonymous.

4a-iii) Information giving during recruitment

When interested employees contacted the study team, they were provided additional information about the study, and their eligibility to participate (that is, age and employment at the company) was confirmed. "Employees interested in participating after learning more about the project provided the study team with an email address to be used to send a personalized email and link to the online baseline survey, which also included the consent document. After reading the consent document, participants selected one of two responses, indicating whether they consented or declined to participate. Participants were not able to continue with the survey until they acknowledged and indicated that they consented to participate."

4b) CONSORT: Settings and locations where the data were collected

"The 30-45 minute online self-report survey contained the following measures..."
"...all employees 50 years of age and older (approximately 2,500 employees) located in multiple U.S. offices of a global information technology company."

4b-i) Report if outcomes were (self-)assessed through online questionnaires

"The 30-45 minute online self-report survey contained the following measures..."

4b-ii) Report how institutional affiliations are displayed

The recruitment materials and informed consent document identified study investigators as "ISA Associates," a research organization.

5) CONSORT: Describe the interventions for each group with sufficient details to allow replication, including how and when they were actually administered

5-i) Mention names, credential, affiliations of the developers, sponsors, and owners
"Conflicts of Interest: The HealthyPast50 program tested in this study is owned by ISA Associates, Inc., a company of which the two senior authors are owners."

5-ii) Describe the history/development process
"The web-based HealthyPast50 program was developed by our group over a two-year period through multiple cycles of development and testing, beginning with focus groups of older workers (age 50+) providing feedback on specific features of the planned program, followed by ratings of prototype content, and culminating in the workplace-based RCT."

5-iii) Revisions and updating
"With the exception of the project update, the program was unchanged throughout the test period."

5-iv) Quality assurance methods
"The program functions were monitored throughout the test period to ensure quality of operation."

5-v) Ensure replicability by publishing the source code, and/or providing screenshots/screen-capture video, and/or providing flowcharts of the algorithms used
Program modules and major elements are described in detail, and screen shots, capture video segments and content outline are presented in appedices and imbedded in the text. No source code is included, but readers are asked to contact authors for access to the program.

5-vi) Digital preservation
The URL and author's email address are archived on WebCite.

5-vii) Access
Participants in the program group could access the web-based program at any time during the three-month test period, both at work or outside work (e.g., at home).

5-viii) Mode of delivery, features/functionalities/components of the intervention and comparator, and the theoretical framework
Users were encouraged to complete "assessments before accessing the other modules. These recommendations were the only form of tailoring used in the program. With the exception of the module on healthy aging, all the major modules incorporated material from previously developed and tested programs from our group (for which evidence of efficacy was shown), modifying the content and approaches for the 50+ audience [19, 22-23]. The program functions were monitored throughout the test period to ensure quality of operation. As with these previous web-based interventions, HealthyPast50 was shaped by a social-cognitive conceptual model based mainly on the work of Bandura [24-25], emphasizing the boosting of self-efficacy, self regulation and planning. A central premise of this model is that in order to achieve lasting improvements in health behavior, an intervention must do more than provide information about a given health topic; it must also provide the skills and motivation that are essential to making lasting improvements in one’s health practices."

5-ix) Describe use parameters
Users were advised to access the program in doses of no more than 30 minutes.

5-x) Clarify the level of human involvement
"The program operated alone and automatically; no human contact was involved."

5-xi) Report any prompts/reminders used
A “project update” email was sent to participants at one-month and two-months post randomization. For the program group, the emails included a reminder to use the program and information about the latest update to the program (a brief message on the home page). With the exception of the project update, the program was unchanged throughout the test period. For the control group, the email included information about when the second survey would be available.'

5-xii) Describe any co-interventions (incl. training/support)
No co-interventions or additional training/support was provided.

6a) CONSORT: Completely defined pre-specified primary and secondary outcome measures, including how and when they were assessed
All measures are described in detail, including their source, validity and reliability.

6a-i) Online questionnaires: describe if they were validated for online use and apply CHERRIES items to describe how the questionnaires were designed/deployed
Virtually measures were previously validated for online use by the authors in previous studies or by other sources.

6a-ii) Describe whether and how “use” (including intensity of use/dosage) was defined/measured/monitored
"Use data, including number of log-ins and pages accessed, were recorded for each member of the program group."

6a-iii) Describe whether, how, and when qualitative feedback from participants was obtained
"The posttest survey sent to the program group also included a 17-item rating form, asking participants to rate, on a five-point response scale, the extent to which they felt the program was accurate, motivating, easy to navigate, helpful in making changes, etc."

6b) CONSORT: Any changes to trial outcomes after the trial commenced, with reasons
No changes to trial outcomes were made after trial commenced.

7a) CONSORT: How sample size was determined

7a-i) Describe whether and how expected attrition was taken into account when calculating the sample size
"The study was powered for a sample size of 250 (after attrition) based on previous studies by the authors."

7b) CONSORT: When applicable, explanation of any interim analyses and stopping guidelines
There were no interim analyses or stopping guidelines.

8a) CONSORT: Method used to generate the random allocation sequence
"Randomization was conducted using a block randomized design with blocks of 4 and 6. The 0 and 1 within each block were random and the order of the group of 4 and the group of 6 was random. Randomization occurred after each participant completed the baseline survey. The online survey program was checked every day to determine who completed the survey each day and individuals were assigned to the next condition on the randomization table as they completed the survey."

8b) CONSORT: Type of randomisation; details of any restriction (such as blocking and block size)
Yes - see 8a.

9) CONSORT: Mechanism used to implement the random allocation sequence (such as sequentially numbered containers), describing any steps taken to conceal the sequence until interventions were assigned
Yes - see 8a.

10) CONSORT: Who generated the random allocation sequence, who enrolled participants, and who assigned participants to interventions
"Randomization was conducted by the second author using a block randomized design with blocks of 4 and 6." The secon author also assigned particpants to conditions.

11a) CONSORT: Blinding - If done, who was blinded after assignment to interventions (for example, participants, care providers, those assessing outcomes) and how

11a-i) Specify who was blinded, and who wasn’t
"Once randomization was complete, participants were notified of the condition in which they were assigned (no blinding procedures were employed)"

11a-ii) Discuss e.g., whether participants knew which intervention was the “intervention of interest” and which one was the “comparator”

Yes - this was a "wait-list" RCT.

11b) CONSORT: If relevant, description of the similarity of interventions

N/A - a "wait list" RCT.

12a) CONSORT: Statistical methods used to compare groups for primary and secondary outcomes

"Data analysis was conducted with SPSS, using analysis of covariance (ANCOVA) to assess program effects on outcome measures and moderator effects, and multiple regression to assess dosage effects. Analyses followed intent-to-treat principles, including all participants irrespective of protocol violations and events arising from post randomization [35]. Imputation was conducted for missing data on all outcome variables, using group mean imputation. Before imputing missing data, we conducted attrition analysis, examining potential baseline differences between those who completed the follow-up survey and those who did not. These data indicated that responders and non-responders were virtually equivalent."

12a-i) Imputation techniques to deal with attrition / missing values

Yes - see 12a above.

12b) CONSORT: Methods for additional analyses, such as subgroup analyses and adjusted analyses

Yes: Analysis of moderator effects (as indicatedin 12a) included subgroup analysis based on gender.

RESULTS

13a) CONSORT: For each group, the numbers of participants who were randomly assigned, received intended treatment, and were analysed for the primary outcome

Yes --
"Two hundred and ninety employees contacted the study team in response to the initial email and were sent a link to the online survey, 279 of whom completed the baseline survey. One participant subsequently contacted the study team to withdraw from the study leaving a total sample size of 278 participants (See Figure 1)."

13b) CONSORT: For each group, losses and exclusions after randomisation, together with reasons

Yes - Consort flow diagram also included as Figure 1.

13b-i) Attrition diagram

Attrition calculated and described, but not in diagram form over time.

14a) CONSORT: Dates defining the periods of recruitment and follow-up

"The data collection started on October, 10, 2012 (first participant enrolled) and ended on February 23, 2013 (last participant completed posttest). Individual access to program by participants was limited to the three-month test period."

14a-i) Indicate if critical “secular events” fell into the study period

"No discernable secular events of note occurred during the test period."

14b) CONSORT: Why the trial ended or was stopped (early)

N/A

15) CONSORT: A table showing baseline demographic and clinical characteristics for each group

Table 1 displays detailed data on sample demographics.

15-i) Report demographics associated with digital divide issues

Although digital divide issues can often be important in ehealth trials, they had little bearing on the results of this trial, as all participants wwere on one side of the divide.

16a) CONSORT: For each group, number of participants (denominator) included in each analysis and whether the analysis was by original assigned groups

16-i) Report multiple “denominators” and provide definitions

Multiple "denominators" and their definitions are reported throughout the manuscript, from consort diagram to text and tables of the Results section.

16-ii) Primary analysis should be intent-to-treat

"Analyses followed intent-to-treat principles, including all participants irrespective of protocol violations and events arising from post randomization."

17a) CONSORT: For each primary and secondary outcome, results for each group, and the estimated effect size and its precision (such as 95% confidence interval)

Statistical values and P levels are shown and discussed throughout the results section.

17a-i) Presentation of process outcomes such as metrics of use and intensity of use

"Data on number of log-ins and number of pages accessed were recorded for all members of the program group. The mean number of log-ins was 2.15 (SD = 3.71, range = 0 – 28), and the mean number of pages viewed was 11.04 (SD = 20.08, range = 0 – 120)." These data weere used in the dosage analysis.

17b) CONSORT: For binary outcomes, presentation of both absolute and relative effect sizes is recommended

Outcomes were continuous and not binary.

18) CONSORT: Results of any other analyses performed, including subgroup analyses and adjusted analyses, distinguishing pre-specified from exploratory

Two types of subgroup analyses were performed: (1) moderator analysis, focused on gender effects; and (2) dosage analysis, assessing program effects by participant use:

"To further explore the nature of the gender X condition interactions, a series of simple effects tests were performed, assessing effects of condition separately for men and women."
"To assess the extent to which the program effects were associated with the extent to which participants in the program group accessed the HealthyPast50 program, regression analysis assessed the relationship between the number of pages viewed and program effects (i.e., dosage effects) for all outcome variables."

18-i) Subgroup analysis of comparing only users

Following intent to treat principles, we analyzed all participants. Non-use in the program group was rare.

19) CONSORT: All important harms or unintended effects in each group

No harm or unintended effects detected or reported.

19-i) Include privacy breaches, technical problems

No privacy breaches or technical problems were encountered.

19-ii) Include qualitative feedback from participants or observations from staff/researchers

"On the 17-item rating form, program group participants gave the program an overall rating of 3.57 on five-point scale. Six of the items received ratings above 4.0; only two items received ratings below 3.0."

DISCUSSION

20) CONSORT: Trial limitations, addressing sources of potential bias, imprecision, multiplicity of analyses

20-i) Typical limitations in ehealth trials

"Although this study exhibited a variety of strengths, including a randomized design, an advanced web-based intervention, and a sizable workforce sample, the study also has some limitations, including – and perhaps foremost – the single posttest at three months and the reliance on self-reports."

21) CONSORT: Generalisability (external validity, applicability) of the trial findings

21-i) Generalizability to other populations
"...because of the particular characteristics of the sample, caution should be exercised in generalizing these findings to workforces that are less educated and affluent. Future research on HealthyPast50 should include longer-term posttests and the inclusion of physical measures – weight, waist circumference, blood pressure, etc. The program also needs to be tested on older workers who are less educated and affluent."

21-ii) Discuss if there were elements in the RCT that would be different in a routine application setting

Because the study was conducted on a workplace sample within a "real life" setting, a routine application would not be appreciably different.

22) CONSORT: Interpretation consistent with results, balancing benefits and harms, and considering other relevant evidence

22-i) Restate study questions and summarize the answers suggested by the data, starting with primary outcomes and process outcomes (use)
Yes: "This randomized trial showed that working adults 50 years of age and older who were given access to the web-based HealthyPast50 program showed significantly greater improvement on key health practices and related constructs over the three month test period than their 50+ counterparts in the control group. Compared to the control group, workers with access to the HealthyPast50 program reported significant improvement in Eating Practices, Self-efficacy for Diet Change, and Healthy Eating Planning."
"The significant (and suggestive) dosage effects found for several measures of diet and exercise indicate that these improvements were associated with the extent to which participants were exposed to the program, and provide further support that the positive changes in participant diet and exercise were a function of the amount of exposure to, and engagement in, the HealthyPast50 program."

22-ii) Highlight unanswered new questions, suggest future research
"Future research on HealthyPast50 should include longer-term posttests and the inclusion of physical measures – weight, waist circumference, blood pressure, etc. The program also needs to be tested on older workers who are less educated and affluent."

Other information

23) CONSORT: Registration number and name of trial registry

Registration of our our trial was not required by NIH.

24) CONSORT: Where the full trial protocol can be accessed, if available

The full trial protocol is included as an appendix.

25) CONSORT: Sources of funding and other support (such as supply of drugs), role of funders

"This research was funded by the National Institute on Aging, Grant Number R44AG033964."

X26-i) Comment on ethics committee approval

All studies by our organization must be approved by our IRB. We have no separate "ethics committee."

x26-ii) Outline informed consent procedures
"Employees interested in participating after learning more about the project provided the study team with an email address to be used to send a personalized email and link to the online baseline survey, which also included the consent document."

X26-iii) Safety and security procedures
As stated in the Consent form: "All data collected online will be done through a secure website. You will use a unique identification number to enter the web-based program and complete the online questionnaires. Your name and contact information will be kept separately from all your other data. You will not enter any personally identifying information when using the program or completing a questionnaire. None of your responses to the questionnaire will be shared with your employer."
Also in the Consent form: "If you have ANY concerns about your health, we recommend that you seek the immediate assistance of a medical or behavioral health care provider such as your physician, family care provider, or an emergency service provider. You may also contact the Employee Assistance Program at 866-808-5062."

X27-i) State the relation of the study team towards the system being evaluated
"Conflicts of Interest: The HealthyPast50 program tested in this study is owned by ISA Associates, Inc., a company of which the two senior authors are owners."
